# Physiological and Proteomic Responses to Drought in Leaves of *Amygdalus mira* (*Koehne*) Yü et Lu

**DOI:** 10.3389/fpls.2021.620499

**Published:** 2021-06-24

**Authors:** Liping Xu, Yanbo Hu, Guangze Jin, Pei Lei, Liqun Sang, Qiuxiang Luo, Zhi Liu, Fachun Guan, Fanjuan Meng, Xiyang Zhao

**Affiliations:** ^1^State Key Laboratory of Tree Genetics and Breeding, Northeast Forestry University, Harbin, China; ^2^College of Life Science, Northeast Forestry University, Harbin, China; ^3^Key Laboratory of Sustainable Forest Ecosystem Management-Ministry of Education, School of Forestry, Northeast Forestry University, Harbin, China; ^4^Tibet Agriculture and Animal Husbandry College, Nyingchi, China; ^5^Department of Medical Genetics, Center for Genome Research, Center for Precision Medicine, Zhongshan School of Medicine, Sun Yat-sen University, Guangzhou, China; ^6^Jilin Academy of Agricultural Science, Changchun, China

**Keywords:** *Amygdalus mira* (*Koehne*) Yü et Lu, physiological, proteomic, drought, stress

## Abstract

Various environmental stresses strongly influence plant development. Among these stresses is drought, which is a serious threat that can reduce agricultural productivity and obstruct plant growth. Although the mechanism of plants in response to drought has been studied extensively, the adaptive strategies of *Amygdalus mira* (*Koehne*) Yü et Lu grown in drought and rewatered habitats remain undefined. *Amygdalus mira* from the Tibetan Plateau has outstanding nutritional and medicinal values and can thrive in extreme drought. In this study, the physiological and proteomic responses in leaves of *A. mira* were investigated during drought and recovery period. The changes in plant growth, photosynthesis, enzymes, and non-enzymatic antioxidant under drought and rewatering were also analyzed in leaves. Compared with controls, *A. mira* showed stronger adaptive and resistant characteristics to drought. In addition, the proteomic technique was also used to study drought tolerance mechanisms in *A. mira* leaves. Differentially expressed proteins were identified using mass spectrometry. Accordingly, 103 proteins involved in 10 functional categories: cytoskeleton dynamics, energy metabolism, carbohydrate metabolism, photosynthesis, transcription and translation, transport, stress and defense, molecular chaperones, other materials metabolism, and unknown function were identified. These results showed that an increase of stress-defense-related proteins in leaves after drought treatment contributed to coping with drought. Importantly, *A. mira* developed an adaptive mechanism to scavenge reactive oxygen species (ROS), including enhancing antioxidant enzyme activities and non-enzymatic antioxidant contents, reducing energy, and adjusting the efficiency of gas exchanges. These results may help to understand the acclimation of *A. mira* to drought.

## Introduction

Drought can decrease the photosynthetic rate in leaves for preventing water loss or affect the capacity of quenching antioxidant and osmotic adjustment of plants (Ahuja et al., 2010; Danquah et al., 2014; Wang and Komatsu, 2017; Wei et al., 2018). At the protein levels, the expression of proteins related to defense and energy generation increased, suggesting that plant defenses and energy consumption were essential for regulating drought (Shinji et al., 2018). For instance, as molecular chaperones, heat shock proteins (HSPs) play critical roles in preserving target protein and helping the recovery of denatured proteins assembly, degradation and translocation of damaged proteins, and protein folding when protecting plants against stress (Wang et al., 2004; Sato and Yokoya, 2008; Timperio et al., 2009; Hochberg et al., 2018). Additionally, inducible defense-related proteins have been described in many plant species upon environmental stress, including drought, salinity, and low temperatures (Lee et al., 2011; Zhou et al., 2016). These proteins serve essential functions in plant life, whether in defense or not. In summary, there are complex responsive mechanisms in plants under drought.

In the Tibetan Plateau, alpine plants are exposed to strong UV radiation, cold, drought, and low oxygen concentration (Ni, 2000; Gou et al., 2007; Ugarte et al., 2019). According to previous studies, alpine plants have evolved various strategies to adapt to this severe situation, including morphology, physiological, and molecular features (Hughes and Atchison, 2015; Zong et al., 2018). In particular, changes in leaf characteristics, such as high levels of enzymatic and non-enzymatic antioxidants, an increase of photosynthetic efficiency, and an increase in pigment accumulation, have been previously reported (Jo et al., 2014; Haiyan et al., 2018). However, the precise physiological and molecular mechanisms of this acclimation are still unclear for alpine plants. In this study, we reported the response of the alpine plant *Amygdalus mira* (*A. mira*) to drought tolerance.

*Amygdalus mira* (*Koehne*) Yü et Lu, which is a deciduous fruit tree, is widespread in mixed forests on hillsides or along valleys of Yunan Province, Sichuan Province, and the Tibetan Plateau, which ranges from 2,000 to 3,400 m in altitude (Zhang et al., 2013; Peng et al., 2015; Cao et al., 2017a). It has outstanding economic, nutritional, and medicinal values. It can thrive in an extreme drought environment. Accordingly, *A. mira* can be used as an excellent resource for drought tolerance-related gene discovery. For this reason, an understanding of the physiological and molecular mechanisms of adaption of *A. mira* to drought stress is fundamental. Thus, to obtain the responsive mechanisms in *A. mira*, systematic experiments should be carried out.

The responses of plants to drought stress have been reported intensively. To withstand water deficiency, plants have developed complex mechanisms, such as reduction of growth, stomatal closure, osmotic adjustment, post-translational modifications, and protein–protein interactions (Chen et al., 2010; Urano et al., 2010; Stéphanie et al., 2011; Meyer et al., 2014). To date, to better understand tolerance mechanisms, the proteomic method is being increasingly performed. Previous investigations on proteome researches of plant species under drought have mainly focused on the model plant but no alpine plants (Urban et al., 2016). As a wild peach species, *A. mira*, unlike cultivated peach, presents higher tolerance to drought (Cao et al., 2017a). Previous studies showed that compared to cultivated peach, *A. mira* displayed contrasting defense characteristics against drought and more sensitive activities of PSII (Bao et al., 2019). Further studies will have solutions for improving cultivated peach.

Some drought tolerance-related proteins have been identified in *Arabidopsis* and *Oryza sativa* until recently (Cao et al., 2017b; Manuka et al., 2018; Nguyen et al., 2018; Saddique et al., 2018; Sylva et al., 2018). The protein regulation networks under drought in many woody plants have also been reported recently (Bonhomme et al., 2009; Simova-Stoilova et al., 2018). Various specialized proteins are identified in plants during drought stress, where they played key and crucial roles as chaperones, signaling molecules, ion homeostasis molecules, osmolytes, reactive oxygen scavengers, and HSPs (Bharathi et al., 2020). The responses of drought-inducible proteins are closely related to plant species. In particular, alpine plants may use special strategies to adapt to the extreme environment, but the underlying proteome and physiological mechanisms of the acclimation need to be better understood.

To our knowledge, unfortunately, there are few studies relating *A. mira* to drought based on proteomics technologies. This study revealed the responding mechanism and identified proteins related to drought responses in *A. mira*. We carried out different physiological measurements on *A. mira* seedlings after drought treatment. Moreover, we also compared the two-dimensional electrophoresis (2-DE) of the leaf proteome of seedlings of *A. mira* under drought.

## Materials and Methods

### Plant Materials and Experimental Design

The experiments were carried out at Northeast Forestry University. All seeds were obtained from the College of Agriculture and Animal Husbandry, Tibet University. The seedlings (1-year-old) were planted in plastic pots containing a 1:3 (v/v) mixture of meteorite and soil. All seedlings were grown for 20 days in a greenhouse. Experiments were divided into two groups: well-watered seedlings were irrigated every 4 days as control (75% field capacity, FC), and for the water deficit treatment (30–50% FC), the irrigation of seedlings was withdrawn for 16 consecutive days until rewatering (day 16). Irrigation was performed every 2 days (regular weighing) to restore the soil moisture at 60–75% FC.

At each time point (days 4, 8, 12, 16, and 20), the leaves of control and treatment seedlings were harvested and stored at −80°C until analysis. Each treatment group was formed with three independent biological replicates.

### Determination of Leaf Water Content and Soil Water Content

Fresh leaves were weighed (FW) immediately and then dried at 70°C until constant weight (DW) was obtained. Leaf water content (LWC) was estimated as follows (Wang et al., 2013). Soil (10 g) from each plastic pot was collected from three randomly selected seedlings, weighed (FWC), and dried at 105°C until constant weight (CWC). Soil water content (SWC) was estimated as demonstrated by Wang et al. (2013).

### Measurement of Gas Exchanges of Leaves

Gas exchange measurements were made on clear and cloudless weather days at 9:00–11:00. Non-detached fully expanded leaves were measured with a Portable Photosynthesis System (LI-6400, LI-COR Inc., Lincoln, NE, United States). The artificial light source was set to 1,200 μmol/m^2^/s, CO_2_ concentration was the atmospheric CO_2_ concentration, relative humidity was 60–70%, airflow rate was 400 μmol/s, and leaf chamber temperature was 28 ± 2°C. Five seedlings per treatment were selected to measure net photosynthesis rate (*P*n), stomatal conductance (*G*s), transpiration rate (*T*r), and intercellular CO_2_ concentration (*C*i).

### Determination of Relative Conductivity, O_2_^−^ and H_2_O_2_

The relative conductivity (REC, %) of leaves was accessed according to the method of Cavalcanti et al. (2007). Four-leaf disks (1 cm^2^) were placed into the tubes containing 20 cm^3^ distilled deionized water. The tubes were incubated at 30°C for 5 h, and the initial electrical conductivity (EC_min_) was determined, and then the tubes were boiled at 100°C for 30 min. After the tubes were cooled to room temperature, the final electrical conductivity (EC_max_) was measured. The REC was calculated: REC (%) = EC_max_/EC_min_ × 100.

Half a gram of fresh leaves was grinded and mixed in a solution containing ethylenediaminetetraacetic acid (EDTA; 0.1 mM), PVP (1%, *w*/*v*), PMSF (0.1 mM), and Triton X-100 (0.2%, *v*/*v*). Then, the mixture was centrifuged at 4,000 *g* for 15 min at 4°C, and the supernatant (1 cm^3^) was mixed with hydroxylamine hydrochloride (1 cm^3^), *β*-aminobenzene sulfonic acid (1 cm^3^), and *α*-naphthylamine (1 cm^3^). Finally, the solution was incubated at 25°C for 20 min. The absorbance of the mixture at 530 nm using a NaNO_2_ standard curve was measured to determine the concentration of superoxide radical (O_2_^−^; Wang et al., 2016a).

The content of H_2_O_2_ was determined by the modified method of Wang et al. (2013). Half a gram of leaves was homogenized in 0.1% cold trichloroacetic acid (TCA; 4 cm^3^) and centrifuged at 4,000 *g* for 10 min. Then, the supernatant (0.5 cm^3^) was put into the mix containing 1 M potassium (1 cm^3^) and potassium phosphate buffer (0.5 cm^3^, 50 mM, pH 6.8). After reaction for 5 min, absorbance values were calculated to the standard curve at 560 nm.

### Assays of SOD, POD, APX, GR, DHAR, and MDHAR

Half a gram of leaves was ground into a fine powder with a mortar and pestle with liquid nitrogen and dissolved in potassium phosphate buffer (10 mM, pH7.0) containing EDTA (1 mM) and polyvinylpyrrolidone (1%). Then, the mixture was centrifuged at 4,000 *g* at 4°C for 30 min.

Superoxide dismutase (SOD; EC1.15.1.1) activity was estimated according to the method of Hernández et al. (1993). The reaction mixture contained 50 mM potassium phosphate buffer (pH 7.8), 195 mM methionine, 0.3 mM EDTA, 1.125 mM NBT, and 60 μM riboflavin. The SOD activity was detected at 560 nm (Beauchamp and Fridovich, 1971).

Peroxidase (POD; EC1.11.1.7) activity was assayed similar to the previous method with minor modifications. The assay mixture contained 2 cm^3^ potassium phosphate buffer (50 mM, pH 7.8), 25 mm^3^ extraction enzyme, 14 mm^3^ guaiacol, and 19 mm^3^ H_2_O_2_ (30%, v/v). POD activity was measured at 470 nm.

Ascorbate peroxidase (APX; EC1.11.1.11) activity assay was measured using the method of Nakano and Asada (1981). The reaction mixture contained potassium phosphate buffer (50 mM, pH 7.8), 2 mM H_2_O_2_, and 200 mm^3^ ascorbic acids (AsA). The APX activity was measured at 290 nm. The enzyme activity of APX was expressed as the degree of oxidation of AsA.

Glutathione reductase (GR; EC1.6.4.2) activity was determined by the method of Carlberg and Mannervik (1975). The reaction mixture contained potassium phosphate buffer (100 mM, pH 7.8), EDTA (2 mM), NADPH (0.2 mM), and glutathione (GSH; 0.5 mM). The GR activity was determined by NADPH oxidation at 340 nm.

For measurement of dehydroascorbate reductase (DHAR) activity (EC 1.8.5.1), the reaction solution contained DHA (0.5 mM) and reduced glutathione (GSH, 5 mM). The DHAR activity was determined at 265 nm according to the method of Dalton et al. (1986).

Monodehydroascorbate reductase (MDHAR; EC 1.6.5.4) activity was determined by the method of Hossain and Asada (1984). The reaction mixture contained NADH (0.2 mM), AsA (1 mM), and AsA oxidase (1 U). The MDHAR activity was measured at 340 nm. One unit of AsA oxidase is equal to the quantity of enzyme that oxidizes 1 mM of AsA to monodehydroascorbate acid per min.

### Measurements of Ascorbate Acid and Glutathione

The content of total ascorbic acid (AsA + DHA) was determined according to the method described by Law et al. (1983). The absorbance was measured at 525 nm, and ASA concentration was obtained according to the standard curve of ASA. Accordingly, total ascorbate (AsA + DHA) was determined. DHA content was calculated from the difference between total ascorbate and AsA.

Total glutathione (the oxidized, GSH + the oxidized, GSSG) were determined according to the method described by Law et al. (1983). The absorbance was measured at 412 nm, and GSH concentration was obtained according to the standard curve of GSH. Statistical analysis of measurement results were obtained using SPSS software.

### Protein Extraction and Quantification

Half a gram of leaves was ground in a fine powder with a mortar and pestle in liquid nitrogen and transferred into the tube contained 10% acetone and 1% polyethylene pyrrolidone. Then the mixture was stored at −20°C overnight. After all, the mixture was centrifugated at 4,000 *g* at 4°C for 20 min. The precipitate was washed with ice-cold 80% then 100% cold acetone and centrifuged at 4,000 *g* at 4°C for 20 min. After centrifugation, the precipitate was vacuum dried. Then, the dried powder was dissolved in lysis buffer [7 M urea, 2 M thiourea, 4% CHAPS, 40 μM DTT, and 0.2 2% (v/v) pH 4–7 IPG buffer]. Protein concentration was determined using Bradford assay using BSA as the standard (Bradford, 1976).

### Gel Electrophoresis, Gel Staining, and Image Analysis

Two-dimensional gel electrophoresis (2-DE) was carried out as described by Wang et al. (2013). About 50 mg of protein samples were used for isoelectric focusing (IEF) 4–7 based on 2-DE gel images. The IEF procedure consisted of the application of 30 V for 14 h, 100 V for 1 h, 500 V for 1 h, 1,000 V for 1 h, 8,000 V for 0.5 h, and 8,000 V for 5 h. After IEF, gels were equilibrated in 10 cm^3^ equilibration buffer I containing 0.1 g DTT for 15 min. Then, gels were incubated in equilibration buffer II containing 1.5 g iodoacetamide instead of DTT for 15 min. The second dimension, SDS-PAGE, was carried out in a 12.5% (w/v) polyacrylamide gel. After electrophoresis, the gels were stained with Coomassie brilliant blue (CBB) R-250 solution.

Gel images were scanned using the Image Scanner III system (GE Healthcare, Bio-Sciences, Uppsala, Sweden) and analyzed by software (Amersham Biosciences, Piscataway, NJ, 2011). The average volume % values were calculated from three technical replicates to represent the final volume % of each biological replicate.

Principal component analysis (PCA) was performed by SPSS version19.0 (SPSS, Inc., Chicago, IL) based on Cheng et al. (2015).

### Matrix-Assisted Time of Flight Mass Spectroscopy Analysis

Protein spots revealed at least 2-fold change between control (optimum) and drought-treated samples at 0.05 level as determined by Student’s *t*-test. Selected protein spots from gels were excised, washed two times with 50% (v/v) acetonitrile in 0.1 M NH_4_HCO_3_, and dried at room temperature. Proteins were reduced with 1 mM DTT and 2 mM NH_4_HCO_3_ at 55°C for 1 h and alkylated with 55 mM iodoacetamide in 25 mM NH_4_HCO_3_ in the dark at room temperature for 45 min. The gel pieces were thoroughly washed with 25 mM NH_4_HCO_3_, 50% acetonitrile, and 100% acetonitrile and dried. The proteins were digested in 10 cm^3^ modified trypsin (Promega, Madison, WI) solution (1 ng/ cm^3^ in 25 mM NH_4_HCO_3_) and incubated overnight at 37°C. Digests were immediately spotted onto 600 mm anchor chips (Bruker Daltonics, Bremen, Germany). Spotting was achieved by pipetting 1 cm^3^ analyte onto the MALDI target plate in duplicate and then adding 0.05 cm^3^ 20 mg/cm^3^ *α*-CHCA in 0.1% TFA/33% (v/v) ACN, which contained 2 mM ammonium phosphate. All samples were analyzed in the positive-ion reflection mode on a time of flight (TOF) Ultraflex II mass spectrometer (Bruker Daltonics, Billerica, MA). Each acquired mass spectra (a m/z range of 700–4,000 and a resolution of 15,000–20,000) were processed using Flex Analysis v2.4 software (Bruker Daltonics, Bremen, Germany, 2004). Based on combined MS and MS/MS spectra, proteins were successfully identified using a 95% or higher CI of their scores in the Mascot V2.3 search engine (Matrix Science Ltd., London, United Kingdom) with the following search Amygdalus parameters: NCBI non-redundant database (release 2019_06; 17,737 sequences; 13,030,327 residues); trypsin as the digestion enzyme; one missed cleavage site; partial modifications of Carbamidomethyl (C) and Oxidation (M); 60 ppm for precursor ion tolerance; and 0.25 Da for fragment ion tolerance.

### Protein Classification and Hierarchical Cluster Analysis

Each protein motifs were classified based on the BLAST alignment, Gene Ontology, and information from the literature. The hierarchical clustering of all proteins was performed according to two change values of protein spots. PCA was employed *via* the website of APPLIED PROTEIN TECHNOLOGY.^1^

### RNA Isolation and Quantitative RT-PCR Analysis

Quantitative real-time PCR (qRT-PCR) was performed to confirm the differential expression of DAPs. Total RNA was isolated from the leaves using a plant RNA extraction kit (Biotecke, China) and reverse-transcribed by PrimeScript Reverse Transcriptase (Takara, Shiga, Japan). The cDNA was amplified in the Lightlycler480 system (Roche, Indianapolis, IN). The gene expression level was detected by SYBR Green Real-time PCR Master Mix (Toyobo, Osaka, Japan). Protein code and forward and reverse primer sequences were listed in Supplementary Table S1. ACTIN was used as the reference gene (Supplementary Table S1). The relative expression level of target genes was calculated using the 2^-△△Ct^ method.

### Statistical Analysis

Statistical analyses were performed with SPSS 17.0 software (SPSS Inc. Chicago, IL2009). All parameters are presented as mean ± SE and were obtained from at least three replicates. Parameters were analyzed using Duncan’s multiple-range test or Student’s *t*-test. A value of *p* < 0.05 was considered significant.

## Results

### Effect of Drought and Rewatering on Plant Growth

**Figure 1:**
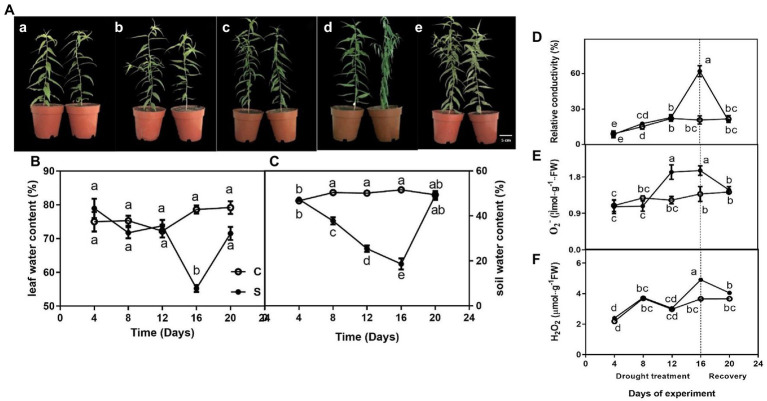
The morphological traits **(Aa–e)** of seedlings leaves during drought treatment (4, 8, 12, and 16 days) and recovery (4 days), leaf water content (LWC; **B**), relative conductivity (REC; **D**), O_2_^−^ **(E)**, and H_2_O_2_ **(F)** of leaves on seedlings growing under control and drought stress, and soil water content (SWC; **C**). The data shown are the mean ± SE of three biological replicates. Different letters indicated significant differences exist at the *p* ≦ 0.05 level.

The leaves of seedlings did not exhibit any obvious phenotype differences on days 4, 8, and 12 of drought (Figures 1Aa–c)., However, seedlings showed wilting on day 16 of drought (Figure 1Ad). Additionally, the morphology of water deficit plants was remarkably improved on day 20 of rewatering (Figure 1Ae).

For control, leaf water content was maintained at approximately 75% but gradually decreased to 20% or so on day 16 of drought and rapidly increased on day 20 of rewatering (Figure 1B). Similarly, drought treatment significantly decreased soil water content on day 16 (Figure 1C).

### Effect of Drought and Rewatering on Relative Conductivity, O_2_^−^ and H_2_O_2_

Relative conductivity of seedlings subjected to drought did not differ significantly compared with controls on days 4, 8, and 12 of treatment, only showed a single peak on day 16 of drought and restored to normal levels after rewatering on day 20 (Figure 1D). Similarly, O_2_^−^·content was significantly higher under drought (on days 12 and 16) than under control conditions and restored to normal levels after rewatering (Figure 1E). The highest value of H_2_O_2_ of leaves on drought-treated seedlings was observed on day 16 when drought was severe compared to control seedlings, whereas this difference was not observed on days 4, 8 and 12 of drought and day 4 of drought rewatering (day 20; Figure 1F).

### Effect of Drought and Rewatering on Photosynthesis

**Figure 2:**
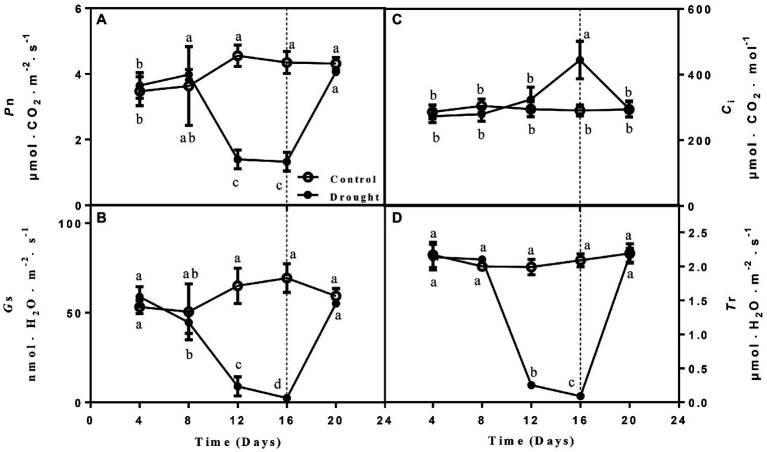
The changes of net photosynthetic rate (*P*n; **A**), stomatal conductance (*G*s; **B**), intercellular CO_2_ concentration (*C*i; **C**), and transpiration rate (*T*r; **D**) of leaves on seedlings growing under control and drought stress and rewatering. The data shown are the mean ± SE of three biological replicates. Different letters indicated significant differences exist at the *p* ≦ 0.05 level.

Four main photosynthetic parameters, including *P*n, *Ci*, *G*s, and *T*r. *P*n, *G*s, and *T*r were measured of the drought treated seedlings gradually decreased on day 12 and 16 compared with control seedlings and increased to normal levels on the 4th day after rewatering. In contrast, *C*i remained higher on days 12 and 16 of drought before declining after rewatering (Figure 2).

### Effect of Drought and Rewatering on Antioxidant Enzymes Activities

**Figure 3:**
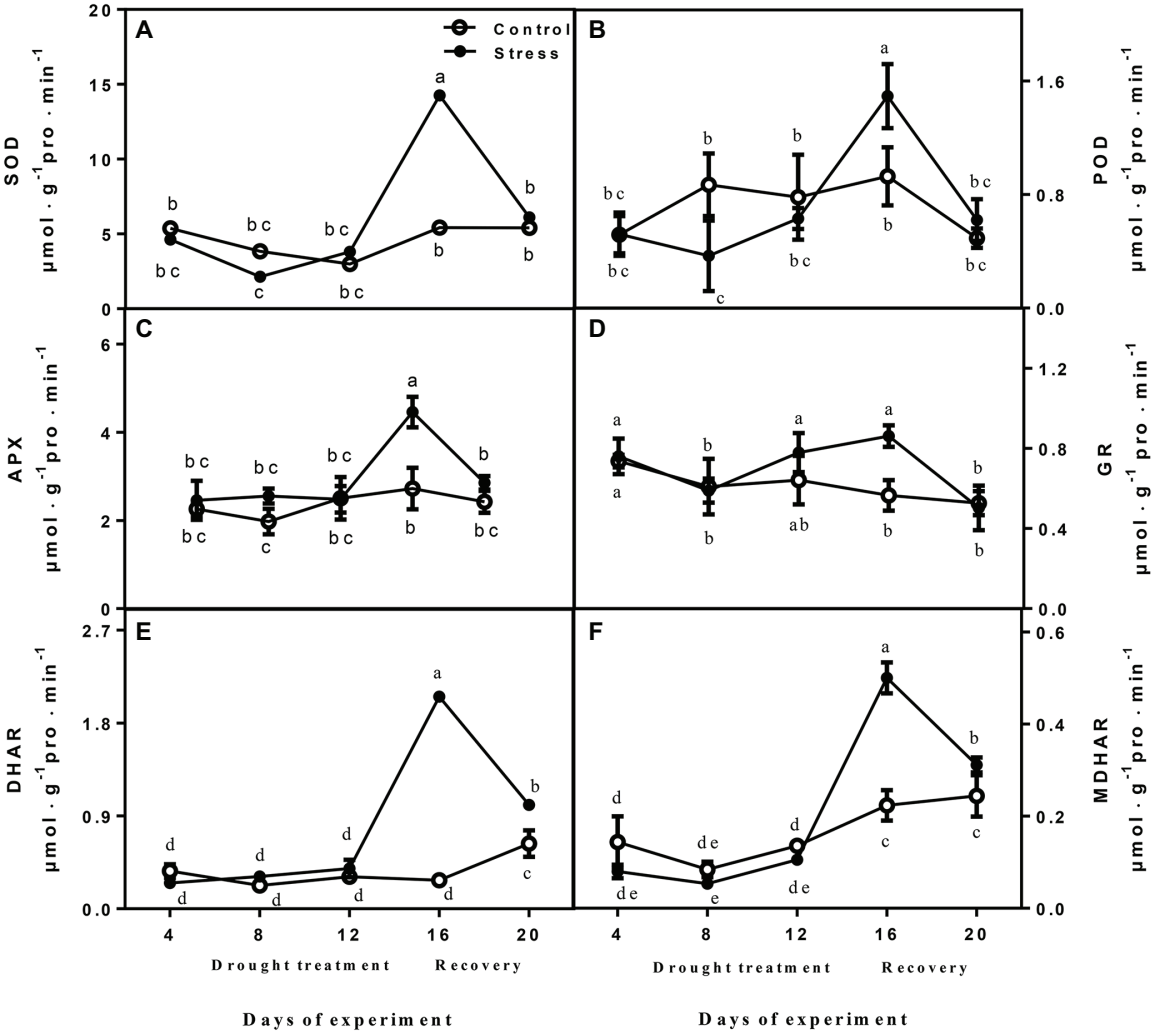
The changes of SOD **(A)**, POD **(B)**, APX **(C)**, GR **(D)**, DHAR **(E)**, and MDHAR **(F)** of leaves on seedlings growing under control and drought stress. The data shown are the mean ± SE of three biological replicates. Different letters indicated significant differences exist at the *p* ≦ 0.05 level. SOD, superoxide dismutase; APX, ascorbate peroxidase; GR, glutathione reductase; DHAR, dehydroascorbate reductase; and MDHAR, monodehydroascorbate reductase.

The activities of SOD, APX, DHAR, GR, and MDHAR did not show obvious variation between drought-treated seedlings and controls at 4, 8, and 12 days, but they were significantly increased after drought treatment (day 16; Figure 3). After rewatering, the parameters were recovered to normal levels, and the activities of APX, DHAR, and MDHAR of drought-treated seedlings were higher than those of controls (Figure 3). POD activity declined rapidly after drought treatment, and the highest value was recorded on day 16, when drought was severe, whereas it decreased upon rewatering (Figure 3).

### Effect of Drought and Rewatering on AsA and GSH

**Figure 4:**
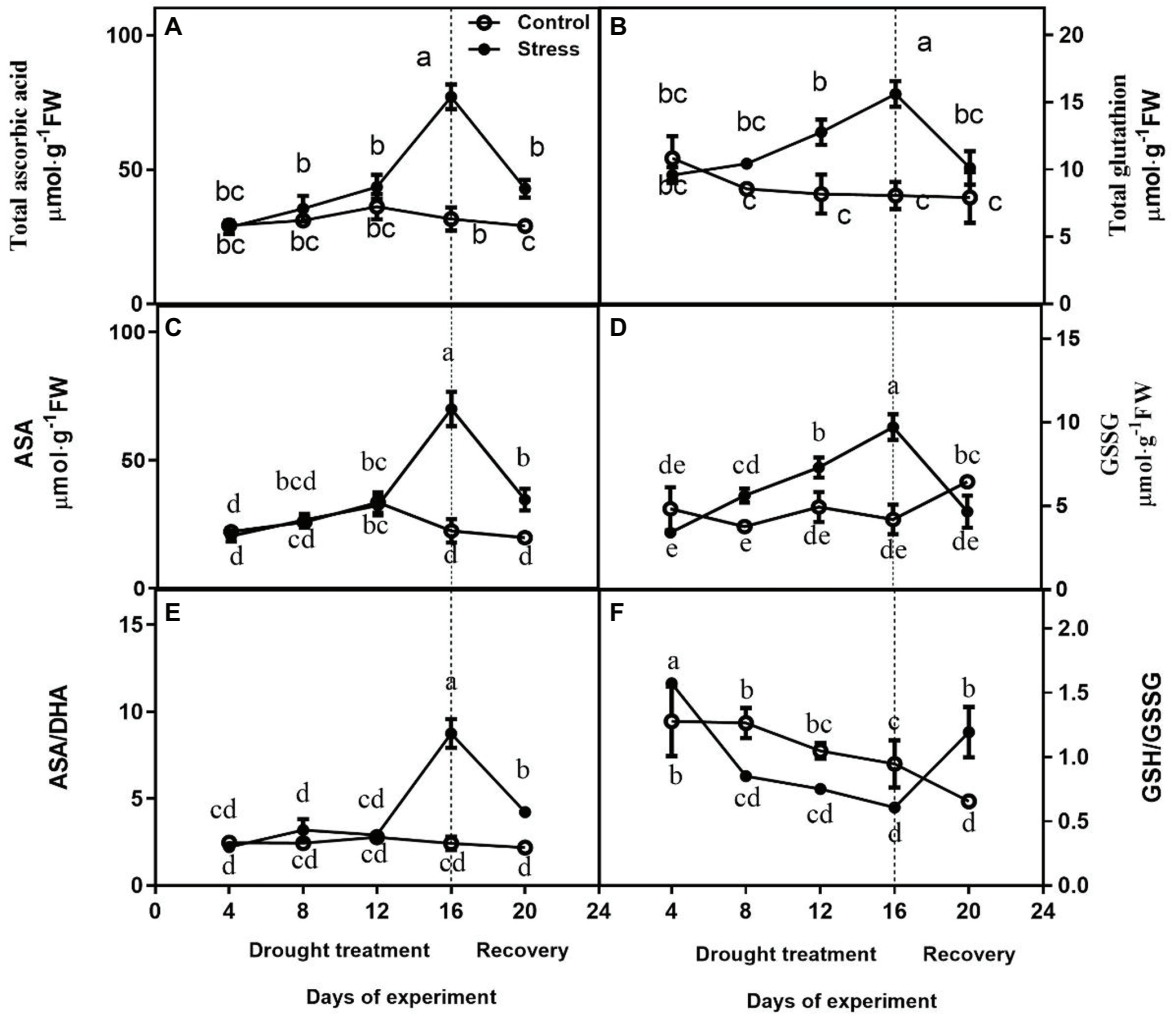
Effects of altitude gradient on the values of total ascorbate content **(A)**, total glutathione content **(B)**, AsA content **(C)**, GSSG content **(D)**, ASA/DHA ration **(E)**, and GSH/GSSG ration **(F)** in leaves under control and drought stress. The data shown are the mean ± SE of three biological replicates. Different letters indicated significant differences exist at the *p* ≦ 0.05 level. AsA, ascorbic acid.

No differences were observed in total ascorbate content, AsA, and ASA/DHA between drought-treated control seedlings at 4, 8, and 12 days, and they were higher under drought than those of controls on day 16 of drought (Figures 4A,C,E). After drought treatment, the Total Glutathione content and GSG content of drought-treated seedlings were significantly higher than that of control seedlings, but GSG content largely decreased upon rewatering (Figures 4B,D). The change of GSH/GSSG was not obvious (Figure 4F).

### Identification of Differentially Abundant Proteins Under Drought and Rewatering

**Figure 5:**
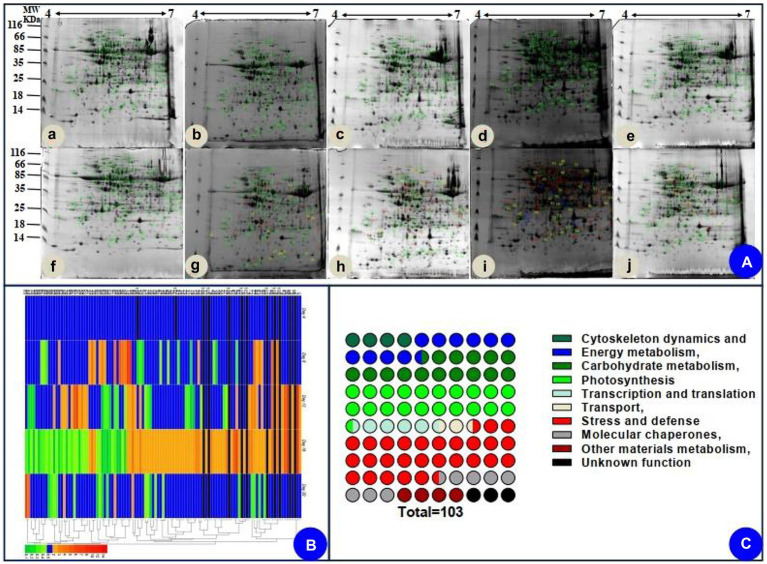
Coomassie brilliant blue-stained two-dimensional electrophoresis (2-DE) gels of proteins **(A)**, hierarchical clustering **(B)**, and functional classification **(C)** of the identified proteins. **(Aa–e)** indicated the electrophoresis gels of protein of seedlings leaves during drought treatment (4, 8, 12, and 16 days) and recovery (4 days). **(Af–j)** indicated the electrophoresis gels of protein of seedlings leaves of watered plants.

Based on the biochemical, physiological, and morphological responses to drought and recovery, we chose five time points to profile drought-responsive and recovery-responsive proteome changes. The image analysis of 2D gels revealed that approximately 1,000 protein spots were detected (Figure 5A), while 103 protein spots were detected as differentially abundant proteins (DAPs; *p* < 0.05). On day 16, 58 of the 103 identified proteins were upregulated, while the rest were downregulated in drought plants relative to their control group. After rewatering, in the drought plants compared with the control group, we found 91 downregulated proteins, five upregulated proteins, and seven proteins that were not significantly differentially expressed in the drought plant in the day 20 group. We chose two-time points, 16 days, which was the longest drought duration, and 20 days, which was during the recovery period after rewatering, to profile drought-responsive and recovery-responsive proteome changes. Comparing the expression of these proteins on day 16 and day 20, 57 proteins were upregulated in the drought plant in day 16 group and downregulated in the drought plant in day 20 group, four proteins were downregulated in the drought plant in day 16 group and upregulated in the drought plant in day 20 group, one protein was upregulated in both the drought plant in day 16 and day 20 group, 33 proteins were downregulated in both the drought plant in day 16 and day 20 group. All these DAPs were submitted for protein identification and PCA analysis. PCA analysis showed that the protein expression trends of drought on day 4 and day 4 after rewatered were consistent (Supplementary Figure S1). Based on the BLAST alignment, Gene Ontology, and information from the literature, 103 DAPs in leaves were classified into 10 functional categories: cytoskeleton dynamics, energy metabolism, carbohydrate metabolism, photosynthesis, transcription and translation, transport, stress and defense, molecular chaperones, other materials metabolism, and unknown function. The information of proteins with different functions is illustrated in Figure 5C, and detailed information of functional classification of all DAPs was listed in Supplementary Tables S2 and S3, respectively. The most represented DAPs in leaves were associated with stress and defense (28.2%), most of which showed an increase on day 16 of drought. Moreover, a total of 21 photosynthesis (20.4%) related DAPs in leaves were identified.

We also assessed how protein level changes during water deficiency and recovery differed at the five time points. As water deficiency progressed and through the recovery period, from day 4 to day 20, there were differences (decreasing or increasing) between control groups and water deficiency groups in protein levels (Supplementary Table S2). In addition, we followed up our analysis of [log2 (fold protein level changes)] in the process of drought and recovery by using κ-means clustering (Figure 5B). The hierarchical clustering analysis was carried out to analyze protein expression characteristics (Figure 5B). All DAPs were clustered into two main groups. A larger number of proteins involved in photosynthesis and stress/defense in the Group I were upregulated on day 16 of drought and recovered to normal levels after rewatering, whereas main proteins in Group II were downregulated clusters on day 16 of drought and recovered to normal levels after rewatering (Figure 5B).

### Leaf Proteomic Characteristics Under Drought and Rewatering

Twenty-one proteins involved in photosynthesis were identified. Chlorophyll a/b binding protein (spots 36 and 37) was induced on day 16 of drought and declined to normal on day 4 after rewatering. CO_2_ assimilation-related proteins, including carbonic anhydrase (Spot 41), ribulose-1,5-bisphosphate carboxylase/oxygenase large subunit (Spot 11), and ribonucleoprotein (Spot 43), were increased on day 16 of drought. In addition, ribulose bisphosphate carboxylase/oxygenase activase, chloroplastic (Spot 98), Photosystem II stability/assembly factor HCF136 (Spot 100), and carbonic anhydrase 2, chloroplastic-like isoform X1 (Spot 102) were also detected only on day 16 of drought.

Twenty-nine stress-defense-related proteins were identified. A slice of enzymatic antioxidants, glutathione S-transferase (GST; spots 39 and 75), peroxiredoxin (Prx; spots 103 and 104), catalase (CAT; spot 32), and abscisic acid stress ripening protein homolog (Asr1; spot 38), were upregulated on day 16 of drought. Several proteins, including arginase (spot 18 and 26) and plastid-lipid-associated protein (spot 30), were also induced after drought treatment (day 16). In contrast, putative glycine-rich RNA-binding protein (spot 66) was upregulated on day 12, and putative luminal binding protein (spot 89) was upregulated on day 4 and downregulated after drought treatment (day 16), respectively.

We also identified 16 proteins involved in carbohydrate metabolism. Among them, 11 proteins were upregulated after drought treatment (day 16). Interestingly, on day 16 after drought treatment, the changes in the abundance of prunasin hydrolase isoform PH B precursor were not uniform: two proteins (spots 76 and 81) increased, but one (spot 90) decreased.

Eleven proteins involved in energy metabolism mainly included ATP synthases (spot 76 and 81) and NADH dehydrogenase (spot 51, 63). These proteins were reduced in drought-treated seedlings on day 16, while two proteins (spots 22 and 72) increased.

Eight proteins involved in molecular chaperones in leaves, including HSP (spot 1.106 and 109), calreticulin (spot 5), endoplasmin homolog (spot 2), endoplasmin-like protein (spot 3), and so on, were found to be remarkably increased by drought treatment (on day 16). The abundance of two proteins related to transport (spots 35 and 107) increased by drought treatment (on day 16). Similarly, three proteins (spot 47, 78, and 79) involved in cytoskeleton dynamics were also induced by drought treatment (on day 16). In addition, three proteins involved in cytoskeleton dynamics reached max levels on day 16 of drought treatment.

### Complementation of DAPs at Transcript Levels by qRT-PCR

**Figure 6:**
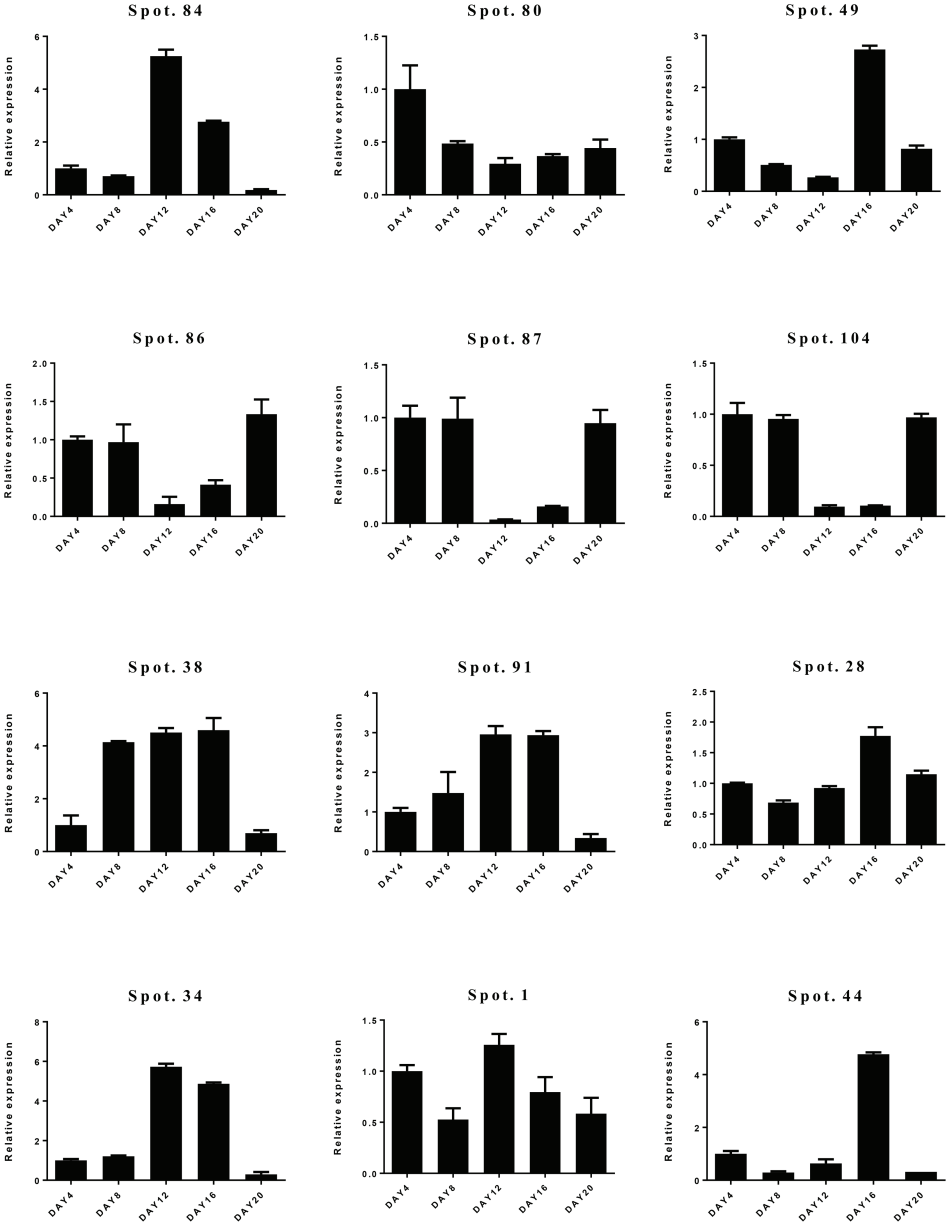
Relative gene expression levels of differentially abundant proteins (DAPs) by quantitative real-time PCR (qRT-PCR).

To confirm the differential expression of DAPs, qRT-PCR was carried out (Figure 6; Supplementary Figure S2). We selected 12 candidate genes representing a variety of functional categories, including cytoskeleton dynamics (spot 84), carbohydrate metabolism (spot 80), transcription and translation (spot 49), stress and defense (spots 28, 34, 38, 86, 87, 91, 104), and molecular chaperones (spot 1). Overall, we found a weak correlation between proteome and qRT-PCR results. Only protein spot (spot 28) involved in stress and defense had consistent expression at transcript and protein levels (Supplementary Table S2; Figure 6).

## Discussion

To adapt to drought, plants used two mechanisms: drought avoidance and drought tolerance (Kooyers, 2015). For drought avoidance, plants often reduce growth to save energy costs and reduce water loss (Zenda et al., 2018). Compared to controls seedlings, growth of *A. mira* seedlings was inhibited after drought treatment. However, a significant recovery of growth can be observed after rewatering, which is also demonstrated that *A. mira* seedlings can rapidly adjust water balance to adapt to water losses. In addition, our proteomics results also revealed that most proteins involved in energy were decreased after drought treatment. This implies that low levels of expression of energy protein contributed to the decline of growth.

To reduce water losses, stoma might be rapidly closed in response to severe drought (McAdam and Brodribb, 2013; Li et al., 2015). Accordingly, *P*n, *G*s, transpiration rates (*T*r) would be inhibited due to stoma closure. When the pores are closed, the carbon dioxide required for photosynthesis cannot enter the tissue where light and action occur, so photosynthesis is weakened. In the present study, photosynthetic parameters including *P*n, *G*s, and *T*r of leaves of *A. mira* seedlings decreased after drought treatment. In contrast to the decline of these parameters upon drought, a significant recovery was detected after rewatering, suggesting that *A. mira* seedlings can enhance growth recovery after drought treatment by adjusting gas exchanges.

Generally, inhibition of photosynthesis would induce reactive oxygen species (ROS), which can lead to oxidative damage in cells (Moller, 2001). To enhance drought tolerance, plants can counteract or scavenge ROS accumulation by antioxidant enzymes and non-enzymatic molecules (Bowler, 1992; Smirnoff, 2000; Mittler, 2002). In the current study, the contents of relative conductivity, O_2_^−^·and H_2_O_2_ of leaves on *A. mira* seedlings were significantly decreased after 16 days of drought (Figure 2). All these changes indicated that drought led to membrane damage. After rewatering, the level of these parameters returned to normal levels. For *A. mira* seedlings, the activities of SOD, APX, DHAR, GR, and MDHAR elevated after drought treatment (16 days), whereas they rose after rewatering. Furthermore, our proteomics results also revealed that the expression levels of three antioxidative enzymes (CAT, GST, and Prx) were increased after drought treatment (Supplementary Table S2). In addition, non-enzymatic antioxidant contents, such as AsA + DHA and GSH + GSSG, were consistently increased after drought treatment. These results demonstrated that a sensitive switch of ROS and active antioxidant capacity between drought and rewatering might be the major mechanism for *A. mira* seedlings to tolerate drought efficiently.

On day 4, 94 proteins were downregulated, while the rest were not significantly different. On day 8, 20 proteins were upregulated, and 76 proteins were downregulated, while the rest were not significantly different. On day 12, 30 proteins were upregulated, and 66 proteins were downregulated, while the rest were not significantly different. The number of upregulated proteins increased significantly in the late stage of drought (day 16). We can see that the number of upregulated proteins was very small in the early stage of drought, which may be due to the mechanism of plant response to drought stress not activated in the early stage of drought, resulting in the protein changes were not obvious.

Principal component analysis was performed to understand the relationship among these treatments as a function of water stress (Supplementary Figure S1). The PCA results suggest this distinction between an early phase of treatment and an advanced phase (8 and 16 days). PCA analyses showed that the leaf proteome was dramatically altered by drought (Supplementary Figure S1). As shown in Supplementary Figure S1, PC1 represented 53.4% of the variance, suggesting differences between day 8 and day 16 in response to the water deficit. Combined with physiological data, the activities of SOD, APX, DHAR, GR, and MDHAR did not show obvious variation at 8 days, but they were significantly increased after drought treatment (day 16; Figure 3). POD activity declined rapidly after drought treatment, and the highest value was recorded on day 16.

To prevent damage and maintain a balance of metabolites, energy is required by plants. In this study, most proteins involved in energy showed lower accumulation after drought treatment (on days 4, 8, 12, and 16). These results suggested that *A. mira* seedlings were able to deal with drought through low energy metabolism. Various processes, such as transporting ions, synthesizing osmolytes, and scavenging ROS, are regulated by energy. Comparative proteomic analyses also showed that when some plant species were subjected to environmental stress, some DAPs are enriched among those involved in energy metabolism processes (Wang et al., 2016b). In contrast, *A. mira* seedlings had different strategies of energy metabolism under drought.

Drought can lead to oxidative damage and ROS production. Accordingly, plants balance oxidative stress by inducing various stress-defense-related proteins. In our experiment, some stress-defense-related proteins were significantly induced by drought (Supplementary Table S2). Especially, two 2-Cys peroxiredoxin proteins (Prx; spots 103 and 105) were detected on day 16 of drought, while the two proteins were not detected on days 4, 8, 12, and 20. It is widely known that peroxiredoxins possess antioxidant capabilities (Rhee-Sue et al., 2012). 2-Cys Prx is a member of the peroxidases and plays an important role in cell protection against oxidative stress by detoxifying peroxides and mediating signaling events (Dietz et al., 2006; Jang et al., 2006). Here, the upregulated expression of 2-Cys Prx suggested that an antioxidant system was induced in leaves of *A. mira* seedlings in response to drought. Two glutathione S-transferase (GSTs; Spot 39 and 75) were also detected after drought treatment. GSTs are multifunctional proteins involved in response to oxidative stress, including drought, heavy metals, salt, and so on (Marrs, 1996; Wagner et al., 2002). It was suggested that GSTs are important to *A. mira* seedlings in stress tolerance. In addition to GSTs, two stress-related protein catalase (CAT; spots 13 and 91) were responsive to the drought treatment. Interestingly, one was decreased, and another was increased in the expression on day 16 of drought. Its function in drought tolerance of *A. mira* seedlings is not clear. Previous research showed that stress-defense-related proteins were significantly regulated in various plant species under drought. Therefore, stress-defense-related proteins should play key roles in response to drought.

Water deficit generally has serious impacts on photosynthesis. These effects can be direct, such as decreased availability of CO_2_ due to limited diffusion through the stomata and the mesophyll or changes in photosynthetic metabolism, or indirectly through oxidative stress. The former is the major determinant of reduced photosynthesis under drought stress in leaves (Chaves et al., 2003). In this study, 21 proteins related to photosynthesis were found to be differentially expressed in the drought. CO_2_ assimilation-related proteins (spots 11, 41, and 43) were upregulated from days 4 to 16. The protein expression reached the highest level on day 16 and then downregulated on day 20. However, it should be noted that some photosystem-related proteins (spots 98, 100, and 102) were downregulated. Meanwhile, photosynthetic parameters including *Pn*, *Gs*, and *Tr* of leaves of *A. mira* decreased obviously after drought (Figure 2). Thus, these different changes of these proteins need to be studied further. We also identified 11 upregulated proteins involved in carbohydrate metabolism after drought treatment (16 days), which suggested the positive acclimation in carbohydrate metabolism of leaves on *A. mira* seedlings to drought. Enhancement of glucose-6-phosphate isomerase (spot 14) and fructokinase (spots 32 and 81) implied that the TCA cycle was increased under drought. Alternatively, ATP will be produced by more carbon substrates. Indeed, our results are consistent with previous proteomic studies.

Here, we found that three HSPs were responsive to drought (Fox et al., 2018). Three HSPs (spots 1, 106, and 109) were increased in abundance after drought treatment. Members of HSPs can help to fold proteins, and assisted folding involves repeated cycles of substrate binding and release according to regulating osmotic stress tolerance (Waters et al., 1996). The increased abundance of HSPs after drought treatment suggests an increase in the transportation of newly synthesized peptides, facilitating intercellular transportation of vital cellular enzymes or maintaining quite a few proteins under drought.

The relationship between protein abundance and gene expression should be analyzed to interpret protein function and stress responses. We found that the abundance of most proteins is not consistent with mRNA levels except for Spot 28. Generally, the relationship between protein and mRNA expression levels attribute to mRNA stability and gene expression regulation. Therefore, a low degree of correlation between transcription and translation to *A. mira* seedlings under drought and rewatering can be due to post-translational regulation. These comparative results also indicated the importance of employing proteomics to reveal tolerance mechanisms to drought.

## Data Availability Statement

The original contributions presented in the study are publicly available. This data can be found at: https://github.com/liuz-bio/ProEPhoreSeqData.

## Author Contributions

LX, XZ, and YH did all the experiments, interpreted the results, and wrote the manuscript. GJ, QL, and FM designed the experiment and performed the technical guidance. FG, ZL, LS, and PL provided the assistance in the data analysis. LX and YH contributed equally to this work. All authors contributed to the article and approved the submitted version.

### Conflict of Interest

The authors declare that the research was conducted in the absence of any commercial or financial relationships that could be construed as a potential conflict of interest.

## Footnotes

^1^http://cloud.aptbiotech.com/
