# Supplementary material for: Physiological and Proteomic Responses to Drought in Leaves of Amygdalus mira (Koehne) Yü et Lu
Source: Front Plant Sci. 2021 Jun 24;12:620499. doi: 10.3389/fpls.2021.620499 (PMC8264794; doi:10.3389/fpls.2021.620499)
Supplement: Supplementary file 1 [file Data_Sheet_1.zip › Figure S2.DOCX]

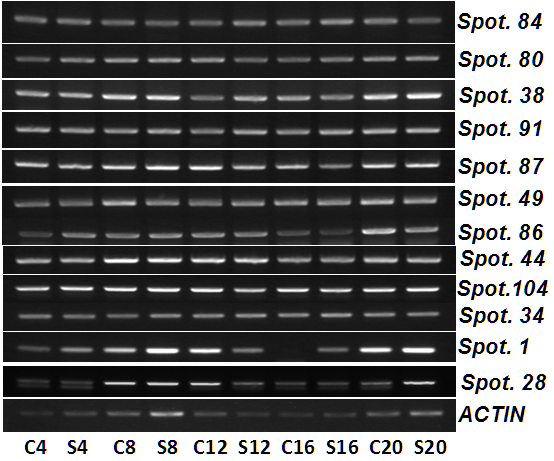


**Fig. S2** Semi-quantitative PCR analysis of *Amygdalus mira (Koehne) Yü et Lu* leaves after drought treatment and re-watering, respectively. C4 and S4, day 4 under control and drought treatment, respectively; C8 and S8, day 8 under control and drought treatment, respectively; C12 and S12, day 12 under control and drought treatment, respectively; C16 and S16, day 16 under control and drought treatment, respectively; C20 and S20, day 4 under control and re-watering, respectively. Data of spots were listed in Supplemental Table S1 and Fig. 5.
