# Supplementary material for: Physiological and Proteomic Responses to Drought in Leaves of Amygdalus mira (Koehne) Yü et Lu
Source: Front Plant Sci. 2021 Jun 24;12:620499. doi: 10.3389/fpls.2021.620499 (PMC8264794; doi:10.3389/fpls.2021.620499)
Supplement: Supplementary file 1 [file Data_Sheet_1.zip › Table S2.DOCX]

**Attached Table S2. Expression levels of differentially expressed proteins in leaves of *Amygdalus mira* (Koehne) Yü et Lu after drought treatment and re-watering**

| **Spot no. ^a^** | **Homologous protein** | **Day 4** | **Day 8** | **Day 12** | **Day 16** | **Day 20** |
| --- | --- | --- | --- | --- | --- | --- |
| **Cytoskeleton dynamics** | | | | | | |
| 78 | actin | 1.815±0.024 | 1.466±0.016 | 0.240±0.009 | 2.122±0.013 | 0.137±0.004 |
| 79 | Full=Actin-1 | 1.764±0.011 | 1.127±0.009 | 0.938±0.014 | 2.107±0.018 | 0.406±0.009 |
| 84 | cell division cycle protein 48, partial | 1.333±0.020 | 0.664±0.017 | 0.254±0.012 | 0.254±0.009 | 4.142±0.038 |
| 47 | hypothetical protein PRUPE_ppa013109mg | 1.864±0.013 | 0.490±0.007 | 1.555±0.013 | 2.266±0.016 | 0.502±0.007 |
| **Energy** | | | | | | |
| 51 | NADH dehydrogenase [ubiquinone] iron-sulfur 1, mitochondrial -like protein | 1.323±0.013 | 0.812±0.011 | 2.315±0.017 | 0.470±0.009 | 1.380±0.013 |
| 53 | D-3-phosphoglycerate dehydrogenase | 1.113±0.020 | 1.731±0.015 | 0.056±0.004 | 0.252±0.009 | 0.517±0.010 |
| 63 | ferredoxin--NADP reductase, leaf isozyme, chloroplastic-like isoform 1 | 1.029±0.013 | 2.014±0.016 | 0.383±0.019 | 0.248±0.010 | 0.861±0.021 |
| 85 | Full=ATP synthase subunit beta, mitochondrial; Flags: Precursor | 1.221±0.011 | 0.918±0.018 | 1.273±0.014 | 0.324±0.011 | 6.436±0.219 |
| 69 | ATP synthase CF1 epsilon subunit | 1.077±0.014 | 0.714±0.017 | 0.512±0.015 | 0.431±0.009 | 0.720±0.012 |
| 94 | hypothetical protein PRUPE_ppa009109mg | 1.075±0.019 | 3.348±0.143 | 0.129±0.012 | 0.043±0.004 | 0.242±0.008 |
| 22 | PREDICTED: protein DJ-1 homolog D-like | 0.950±0.021 | 1.776±0.019 | 0.415±0.024 | 2.699±0.093 | 1.405±0.013 |
| 55 | Os02g0101500 | 0.653±0.052 | 3.856±0.333 | 0.455±0.017 | 0.226±0.011 | 1.091±0.021 |
| 72 | cytosolic ATP sulfurylase | 0.912±0.030 | 1.378±0.017 | 3.322±0.052 | 3.863±0.061 | 0.380±0.009 |
| 93 | hypothetical protein PRUPE_ppa008220mg | 1.587±0.022 | 1.875±0.016 | 0.724±0.021 | 0.422±0.012 | 0.122±0.008 |
| 50 | CLPC | 0.916±0.014 | 1.578±0.021 | 0.323±0.012 | 0.160±0.006 | 1.108±0.027 |
| **Carbohydrate metabolism** | | | | | | |
| 52 | hypothetical protein PRUPE_ppa002686mg | 1.153±0.018 | 0.773±0.013 | 1.359±0.025 | 0.456±0.013 | 1.266±0.017 |
| 75 | prunasin hydrolase isoform PH B precursor | 0.902±0.020 | 1.120±0.014 | 0.385±0.010 | 2.673±0.044 | 0.433±0.013 |
| 76 | prunasin hydrolase isoform PH B precursor | 1.884±0.017 | 0.539±0.015 | 1.008±0.012 | 2.790±0.069 | 0.390±0.011 |
| 81 | fructose-bisphosphate aldolase | 1.220±0.013 | 1.286±0.021 | 0.488±0.011 | 2.054±0.161 | 0.457±0.007 |
| 32 | fructokinase, putative | 1.548±0.021 | 0.695±0.015 | 0.346±0.009 | 2.172±0.132 | 1.283±0.027 |
| 24 | LOS2 | 1.446±0.017 | 0.278±0.013 | 1.659±0.022 | 2.172±0.042 | 1.091±0.013 |
| 16 | PREDICTED: xylulose kinase | 0.688±0.013 | 0.072±0.010 | 0.512±0.010 | 2.482±0.053 | 1.115±0.015 |
| 61 | triosephosphate isomerase | 0.957±0.030 | 0.517±0.013 | 7.462±0.502 | 0.430±0.010 | 1.365±0.021 |
| 56 | hypothetical protein PRUPE_ppa006386mg | 0.696±0.019 | 4.303±0.038 | 0.803±0.013 | 0.448±0.013 | 1.381±0.012 |
| 77 | hypothetical protein PRUPE_ppa003856mg | 1.413±0.015 | 4.222±0.065 | 3.120±0.102 | 2.663±0.072 | 0.329±0.024 |
| 90 | prunasin hydrolase isoform PH B precursor | 1.233±0.018 | 1.729±0.023 | 0.326±0.016 | 0.130±0.009 | 0.297±0.008 |
| 14 | Glucose-6-phosphate isomerase | 1.913±0.013 | 7.281±0.488 | 2.399±0.046 | 2.374±0.033 | 1.672±0.025 |
| 80 | sedoheptulose-1,7-bisphosphatase, chloroplastic-like | 0.879±0.013 | 1.363±0.013 | 1.405±0.020 | 2.141±0.033 | 0.331±0.015 |
| 23 | ADP-glucose pyrophosphorylase alpha subunit IbAGPa2 | 1.531±0.024 | 1.029±0.015 | 0.348±0.011 | 2.423±0.032 | 1.199±0.021 |
| 54 | mandelonitrile glucosyltransferase UGT85A19 | 1.175±0.017 | 1.070±0.006 | 2.182±0.023 | 0.416±0.016 | 1.069±0.017 |
| 83 | Ferredoxin-thioredoxin reductase catalytic chain, chloroplastic -like protein | 0.630±0.013 | 3.127±0.033 | 1.908±0.017 | 2.810±0.026 | 0.346±0.010 |
| **Photosynthesis** | | | | | | |
| 36 | putative chlorophyll a/b binding protein, partial | 1.636±0.025 | 1.258±0.018 | 1.126±0.013 | 2.581±0.022 | 0.988±0.018 |
| 58 | 28 kDa ribonucleoprotein, chloroplastic-like | 1.850±0.017 | 1.282±0.010 | 3.390±0.037 | 0.421±0.011 | 1.289±0.021 |
| 60 | carbonic anhydrase 2, chloroplastic-like isoform X1 | 1.152±0.013 | 0.310±0.011 | 0.544±0.007 | 0.446±0.013 | 0.627±0.013 |
| 65 | PREDICTED: gamma carbonic anhydrase 1, mitochondrial | 1.193±0.021 | 2.057±0.028 | 1.806±0.019 | 0.257±0.013 | 0.824±0.014 |
| 52 | ribulose-1,5-bisphosphate carboxylase/oxygenase large subunit | 0.987±0.016 | 0.704±0.010 | 0.273±0.012 | 0.145±0.008 | 0.892±0.012 |
| 70 | ribulose-1,5-bisphosphate carboxylase/oxygenase large subunit | 0.959±0.021 | 0.604±0.011 | 0.278±0.009 | 0.175±0.006 | 0.692±0.013 |
| 98 | Ribulose bisphosphate carboxylase/oxygenase activase, chloroplastic |  |  |  | Only |  |
| 100 | Photosystem II stability/assembly factor HCF136, chloroplastic; Flags: Precursor |  |  |  | Only |  |
| 37 | Full=Chlorophyll a-b binding protein of LHCII type I, chloroplastic | 1.246±0.022 | 2.367±0.020 | 1.002±0.017 | 2.274±0.025 | 1.404±0.013 |
| 97 | Thylakoid lumenal 15 kDa protein, chloroplast precursor, putative | 1.480±0.014 | 0.618±0.013 | 5.507±0.101 | 0.475±0.017 | 0.421±0.014 |
| 64 | ribulose-1,5-bisphosphate carboxylase/oxygenase large subunit, partial (chloroplast) | 1.619±0.017 | 1.491±0.021 | 0.557±0.025 | 0.108±0.010 | 1.333±0.027 |
| 41 | Carbonic anhydrase, chloroplastic | 0.882±0.018 | 0.571±0.014 | 1.663±0.012 | 5.887±0.212 | 1.329±0.017 |
| 40 | D-Ribulose-5-phosphate 3-epimerase | 1.397±0.015 | 0.586±0.015 | 2.740±0.047 | 2.765±0.039 | 1.479±0.011 |
| 59 | carbonic anhydrase 2 | 0.530±0.019 | 5.648±0.162 | 0.820±0.016 | 0.173±0.013 | 0.975±0.023 |
| 43 | Ribonucleo protein At2g37220, chloroplastic | 1.160±0.017 | 0.661±0.024 | 5.419±0.082 | 5.758±0.091 | 0.604±0.013 |
| 11 | ribulose-1,5-bisphosphate carboxylase/oxygenase large subunit, partial (chloroplast) | 0.600±0.021 | 1.716±0.021 | 1.351±0.017 | 2.893±0.030 | 1.026±0.014 |
| 57 | Photosystem II stability/assembly factor, chloroplast (HCF136) isoform 1 | 1.493±0.015 | 1.554±0.013 | 2.616±0.026 | 0.242±0.010 | 1.354±0.013 |
| 29 | 31 kDa ribonucleoprotein | 1.680±0.011 | 0.562±0.016 | 1.322±0.011 | 2.397±0.028 | 0.521±0.009 |
| 74 | photosystem II oxygen-evolving complex protein 2 precursor | 0.626±0.014 | 0.669±0.013 | 2.389±0.023 | 0.281±0.010 | 1.447±0.017 |
| 82 | 23 kDa OEC protein, partial | 0.967±0.020 | 0.932±0.015 | 1.145±0.017 | 2.692±0.039 | 0.342±0.014 |
| 102 | carbonic anhydrase 2, chloroplastic-like isoform X1 |  |  |  | Only |  |
| **Transcription and translation** | | | | | | |
| 21 | eukaryotic initiation factor 4A | 0.651±0.017 | 0.671±0.014 | 0.327±0.011 | 2.142±0.031 | 1.009±0.014 |
| 95 | PREDICTED: 28 kDa ribonucleoprotein, chloroplastic | 1.117±0.033 | 0.719±0.016 | 1.048±0.023 | 0.390±0.014 | 0.309±0.011 |
| 67 | eIF5A3 | 1.389±0.014 | 0.441±0.010 | 0.645±0.021 | 0.384±0.012 | 0.333±0.012 |
| 49 | eukaryotic translation initiation factor 5A | 0.995±0.014 | 0.411±0.017 | 0.612±0.021 | 2.588±0.026 | 1.550±0.018 |
| 4 | OSJNBa0091D06.15 | 0.734±0.015 | 1.637±0.020 | 2.166±0.012 | 2.202±0.018 | 1.139±0.013 |
| **Transport** | | | | | | |
| 107 | hypothetical protein CICLE_v10022955mg |  | 0.555±0.022 | 0.910±0.019 | 2.399±0.027 | 0.900±0.014 |
| 35 | isopentenyl pyrophosphate:dimethyllallyl pyrophosphate isomerase | 1.952±0.011 | 1.515±0.017 | 0.508±0.012 | 2.472±0.025 | 1.040±0.015 |
| **Stress and defense** | | | | | | |
| 30 | plastid-lipid-associated protein, chloroplastic-like | 1.982±0.022 | 1.101±0.016 | 0.679±0.016 | 2.263±0.032 | 0.581±0.019 |
| 86 | PREDICTED: probable glutathione S-transferase | 1.108±0.017 | 0.714±0.021 | 2.822±0.025 | 0.490±0.013 | 2.394±0.017 |
| 87 | thioredoxin peroxidase, partial | 1.652±0.024 | 4.754±0.183 | 0.375±0.020 | 0.326±0.016 | 2.502±0.023 |
| 92 | PREDICTED: universal stress protein A-like protein | 1.947±0.014 | 1.129±0.017 | 2.302±0.014 | 0.097±0.009 | 0.403±0.011 |
| 104 | peroxiredoxin |  |  |  | Only |  |
| 88 | hypothetical protein PRUPE_ppa012662mg | 0.950±0.014 | 0.872±0.019 | 3.079±0.010 | 4.579±0.147 | 2.359±0.025 |
| 38 | abscisic acid stress ripening protein homolog | 0.976±0.015 | 0.913±0.019 | 1.125±0.014 | 2.780±0.028 | 1.505±0.019 |
| 101 | putative lactoylglutathione lyase-like isoform X1 |  |  |  | Only |  |
| 32 | abscisic stress ripening protein homolog | 1.548±0.023 | 0.695±0.017 | 0.346±0.022 | 2.172±0.015 | 1.283±0.015 |
| 91 | catalase | 1.637±0.016 | 2.318±0.027 | 1.560±0.013 | 0.442±0.016 | 0.439±0.011 |
| 13 | Full=Catalase isozyme 2 | 1.337±0.020 | 9.699±0.262 | 2.557±0.034 | 2.918±0.072 | 0.877±0.019 |
| 39 | glutathione S-transferase, amino-terminal domain protein | 1.099±0.012 | 2.276±0.031 | 1.008±0.011 | 3.150±0.019 | 1.353±0.012 |
| 75 | glutathione S-transferase, amino-terminal domain protein | 0.902±0.017 | 1.120±0.019 | 0.385±0.010 | 2.673±0.025 | 0.433±0.014 |
| 45 | hypothetical protein PRUPE_ppa011383mg | 1.872±0.014 | 0.533±0.013 | 1.245±0.028 | 2.866±0.023 | 1.429±0.021 |
| 28 | hypothetical protein PRUPE_ppa008881mg | 1.166±0.012 | 0.357±0.012 | 2.106±0.011 | 7.575±0.173 | 0.656±0.017 |
| 34 | hypothetical protein PRUPE_ppa009612mg | 1.960±0.025 | 0.306±0.013 | 2.419±0.017 | 3.540±0.068 | 1.403±0.020 |
| 31 | quinone oxidoreductase-like family protein | 1.104±0.019 | 2.200±0.023 | 2.495±0.022 | 2.767±0.028 | 0.509±0.015 |
| 105 | PREDICTED: 2-Cys peroxiredoxin BAS1, chloroplastic |  |  |  | Only |  |
| 20 | hypothetical protein PRUPE_ppa011383mg | 1.474±0.021 | 2.073±0.019 | 1.254±0.013 | 2.149±0.015 | 1.046±0.013 |
| 48 | hypothetical protein PRUPE_ppa012591mg | 1.974±0.018 | 0.413±0.014 | 0.739±0.023 | 2.206±0.016 | 1.293±0.014 |
| 103 | 2-Cys peroxiredoxin, partial |  |  |  | Only |  |
| 46 | hypothetical protein PRUPE_ppa012585mg | 1.101±0.016 | 4.684±0.085 | 4.337±0.107 | 4.845±0.092 | 1.731±0.017 |
| 89 | putative luminal binding protein | 1.730±0.023 | 0.729±0.017 | 1.310±0.018 | 0.455±0.014 | 0.358±0.011 |
| 66 | putative glycine-rich RNA-binding protein | 0.743±0.015 | 0.765±0.022 | 2.113±0.017 | 0.319±0.010 | 0.733±0.024 |
| 17 | PREDICTED: 2-hydroxyacyl-CoA lyase | 1.670±0.021 | 0.260±0.008 | 0.023±0.003 | 2.672±0.026 | 1.233±0.019 |
| 26 | arginase 2 | 1.461±0.017 | 0.764±0.017 | 7.222±0.047 | 7.599±0.065 | 1.501±0.019 |
| 18 | arginase 2 | 0.746±0.019 | 1.188±0.014 | 0.767±0.023 | 2.284±0.022 | 1.037±0.019 |
| 33 | hypothetical protein PRUPE_ppa009612mg | 1.712±0.023 | 4.256±0.028 | 0.893±0.015 | 3.259±0.037 | 0.584±0.022 |
| 31 | PREDICTED: ran-binding protein 1 homolog c-like | 1.104±0.020 | 2.200±0.026 | 2.495±0.029 | 2.767±0.019 | 0.509±0.016 |
| **Molecular chaperones** | | | | | | |
| 2 | PREDICTED: endoplasmin homolog | 1.445±0.022 | 1.493±0.018 | 13.913±0.212 | 11.703±0.137 | 1.507±0.019 |
| 106 | Full=17.8 kDa class I heat shock protein |  | 0.290±0.018 | 1.500±0.030 | 2.275±0.027 | 1.598±0.015 |
| 109 | small molecular heat shock protein 19 |  |  |  | Only |  |
| 1 | PREDICTED: heat shock 70 kDa protein 15-like | 1.426±0.018 | 1.009±0.013 | 2.117±0.020 | 2.143±0.018 | 1.348±0.014 |
| 3 | Endoplasmin-like protein | 0.726±0.014 | 0.573±0.017 | 2.163±0.021 | 2.554±0.019 | 0.964±0.017 |
| 9 | Os01g0840100 | 0.615±0.016 | 1.918±0.026 | 1.696±0.019 | 3.080±0.037 | 1.972±0.021 |
| 5 | RecName: Full=Calreticulin; Flags: Precursor | 1.199±0.023 | 0.759±0.017 | 2.289±0.021 | 3.261±0.028 | 0.599±0.015 |
| 10 | luminal binding protein (BiP) | 0.863±0.025 | 0.788±0.019 | 0.771±0.014 | 2.397±0.022 | 1.019±0.013 |
| **Other materials metabolism** | | | | | | |
| 7 | Full=(R)-mandelonitrile lyase 4 | 0.849±0.018 | 1.636±0.014 | 1.579±0.013 | 2.325±0.025 | 0.546±0.014 |
| 44 | allene oxide cyclase | 1.446±0.021 | 2.300±0.026 | 2.233±0.023 | 2.289±0.021 | 0.705±0.014 |
| 39 | PREDICTED: peptide methionine sulfoxide reductase A1-like | 1.077±0.017 | 0.714±0.017 | 0.512±0.010 | 0.431±0.013 | 0.720±0.019 |
| 96 | hypothetical protein PRUPE_ppa011916mg | 1.461±0.014 | 0.756±0.016 | 1.491±0.017 | 0.316±0.012 | 0.259±0.012 |
| **Unknown function** | | | | | | |
| 19 | uncharacterized protein LOC100798637 | 1.252±0.022 | 0.497±0.010 | 0.059±0.009 | 2.644±0.028 | 0.875±0.013 |
| 62 | hypothetical protein PRUPE_ppa008139mg | 0.700±0.013 | 1.794±0.020 | 2.324±0.019 | 0.362±0.011 | 0.597±0.013 |
| 73 | APE1 | 1.191±0.026 | 0.866±0.018 | 0.282±0.012 | 0.483±0.012 | 0.739±0.015 |

^a^ Spot number indicated in Fig. 5; DAY 4, 4 days after drought treatment; DAY 8, 8 days after drought treatment; DAY 12, 12 days after drought treatment; DAY 16, 16 days after drought treatment; DAY 20, 20 days after re-watering; Only indicted those proteins detected only at day 16 of drought treatment; Data indicated Mean of relative protein abundance ± SE (standard error).
