# Supplementary material for: Physiological and Proteomic Responses to Drought in Leaves of Amygdalus mira (Koehne) Yü et Lu
Source: Front Plant Sci. 2021 Jun 24;12:620499. doi: 10.3389/fpls.2021.620499 (PMC8264794; doi:10.3389/fpls.2021.620499)
Supplement: Supplementary file 1 [file Data_Sheet_1.zip › Table S1.DOCX]

Table S1 The primer sequences for Real-time PCR.

| Spot No. | Forward (5'-3') | Reverse (5'-3') | Tm  (°C) | Cycle Number (n) |
| --- | --- | --- | --- | --- |
| *ACTIN* | ATTGTGAGCAACTGGGATG | CTGACACCATCTCCAGAGTC | 53 | 32 |
| Spot.84 | CCTTGTCAGAGGAGGAATGAG | AACACCACCCACATCATCATA | 56 | 40 |
| Spot.80 | TCTCTCCTGCTTCCATCTCT | CCACAAGATGCTGTCCTCA | 56 | 40 |
| Spot.49 | CGGAGCTTCCAAGACCTA | GGAACATCACAATTGTGGG | 55 | 40 |
| Spot.86 | TTGTTGGATTTCTGGCCC | AGTCAGCCCAGAACCTGG | 55 | 40 |
| Spot.87 | TGTGATTCTGTTTTTCTACCCA | ATGAAAAGTCCTCTCAATGCAA | 54 | 40 |
| Spot.104 | TTGGGTGTTTCAGTTGACAG | ACATACTGCAAGGCCTGGA | 55 | 40 |
| Spot.38 | TCTTCCACCACCACAAGGA | GGTGCTTCTCCTCCTTCTTG | 56 | 40 |
| Spot.91 | CGTGTTGTCCATGCTAGGG | CCCTCTCTGGTGTAAAACTTC | 56 | 40 |
| Spot.28 | CAAGGATTTGAAGGCTGAT | CCACAAGAAACTTCTCATAGTCTC | 55 | 40 |
| Spot.34 | GATGTTCGGTTGTCTGTTCG | TTTTCCTCAACTTCCTCTGG | 55 | 40 |
| Spot.1 | GGGGTATTGACGTTGTGCT | GCATTCCAAGAACTTGGGTA | 55 | 40 |
| Spot.44 | GACCCACAAAAGTTCAAGAA | AGTCTCCGAAGTAGAAGCTGT | 53 | 40 |
