# Supplementary figures and images for: Physiological and Proteomic Responses to Drought in Leaves of Amygdalus mira (Koehne) Yü et Lu
Source: Front Plant Sci. 2021 Jun 24;12:620499. doi: 10.3389/fpls.2021.620499 (PMC8264794; doi:10.3389/fpls.2021.620499)

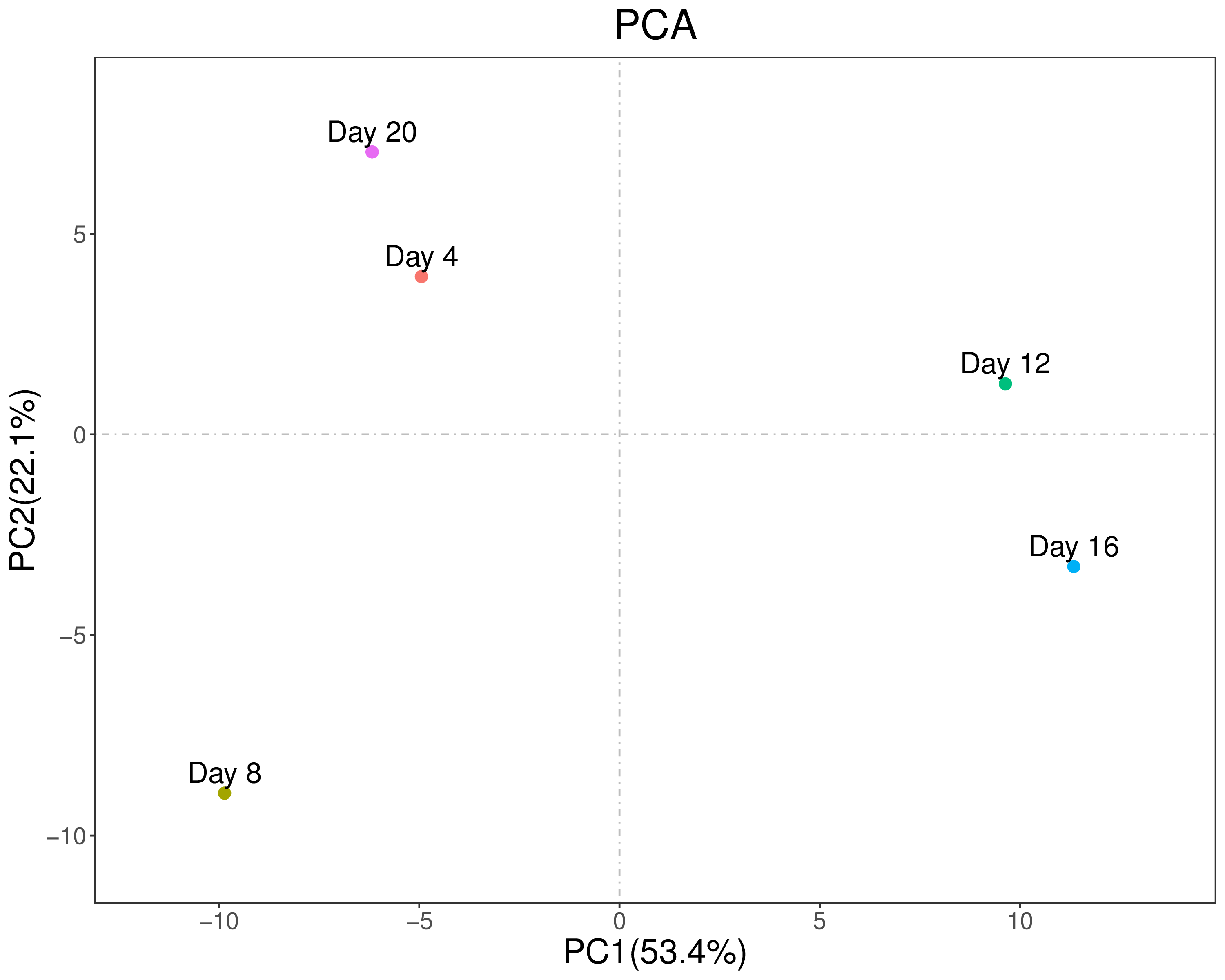


**Fig. S1** Principal component analysis (PCA) of the expressed proteins from the samples.

Supplement: Supplementary file 1 [file Data_Sheet_1.zip › Figure S1.DOCX]
