# Supplementary material for: Physiological and Proteomic Responses to Drought in Leaves of Amygdalus mira (Koehne) Yü et Lu
Source: Front Plant Sci. 2021 Jun 24;12:620499. doi: 10.3389/fpls.2021.620499 (PMC8264794; doi:10.3389/fpls.2021.620499)
Supplement: Supplementary file 1 [file Data_Sheet_1.zip › Table S3.DOC]

**Attached Table S3. Differentially expressed proteins in leaves of *Amygdalus mira* (Koehne) Yü et Lu by mass spectrometry (MS) analysis**

| **Spot no. ^a^** | **Homologous protein** | **Species** | **Accession ^b^** | **Theor p*I*/Mr ^c^** | **Exper p*I*/Mr ^d^** | **score ^e^** | **C (%) ^f^** | **PN ^j^** | **Peptide sequence** |
| --- | --- | --- | --- | --- | --- | --- | --- | --- | --- |
| **Cytoskeleton dynamics** | | | | | | | | | |
| 78 | actin | *Gossypium hirsutum* | 32186898 | 5.44/42 | 5.51/43 | 802 | 34 | 8 | K.AGFAGDDAPR.A R.GYMFTTTAER.E K.GEYDESGPSIVHR.K K.IWHHTFYNELR.V + Dioxidation (W) K.NYELPDGQVITIGAER.F R.VAPEEHPVLLTEAPLNPK.A K.DLYGNIVLSGGSTMFPGIADR.M + Oxidation (M) R.TTGIVLDSGDGVSHTVPIYEGYALPHAILR.L |
| 79 | Full=Actin-1 | *Daucus carota* | 113217 | 5.64/42 | 5.56/43 | 117 | 7 | 2 | R.IWHHTFYNELR.A + Dioxidation (W) K.NYELPDGQVITIGAER.F |
| 84 | cell division cycle protein 48, partial | *Zea mays* | 336093313 | 5.21/86 | 5.38/89 | 443 | 12 | 7 | R.ELVELPLR.H K.DFSTAILER.K K.TALGTSNPSALR.E R.EIDIGVPDEVGR.L R.LDQLIYIPLPDEQSR.L + Deamidated (NQ) K.YTQGFSGADITEICQR.A K.DTHGYVGADLAALCTEAALQCIR.E |
| 47 | hypothetical protein PRUPE_ppa013109mg | *Prunus persica* | 595830675 | 6.60/16 | 6.60/17 | 291 | 61 | 6 | R.IFFIAWSPDTSR.V R.IFFIAWSPDTSR.V + Dioxidation (W) M.ANAASGMAVHDDCK.L + Acetyl (Protein N-term); Oxidation (M) R.YAVFDFDFLTPEGVQK.S K.QVVVESVGEPTETYEQFAGK.L R.ELDGIQIELQATDPTEIGLDVIK.S |
| **Energy** | | | | | | | | | |
| 51 | NADH dehydrogenase [ubiquinone] iron-sulfur 1, mitochondrial -like protein | *Gossypium arboreum* | 728834776 | 6.05/81 | 6.02/74 | 211 | 5 | 3 | R.LPYDTLGAIR.S  K.NPAIIVGAGLFER.D  K.GTETIDVTDAVGSNIR.I |
| 53 | D-3-phosphoglycerate dehydrogenase | *Pinus pinaster* | 346983237 | 8.00/69 | 6.27/59 | 82 | 3 | 2 | K.LGEAGLKVLR.T  R.GGVIDDEALVR.A |
| 63 | PREDICTED: ferredoxin--NADP reductase, leaf isozyme, chloroplastic-like isoform 1 | *Glycine max* | 356502850 | 8.38/40 | 6.84/35 | 114 | 11 | 3 | K.DPNATIIMLGTGTGIAPFR.S + Oxidation (M)  K.ITGDDAPGETWHMVFSTEGEIPYR.E  K.ITGDDAPGETWHMVFSTEGEIPYR.E + Dioxidation (W); Oxidation (M) |
| 85 | Full=ATP synthase subunit beta, mitochondrial; Flags: Precursor | *Nicotiana plumbaginifolia* | 114421 | 5.95/59 | 5.35/49 | 617 | 15 | 6 | K.VVDLLAPYQR.G K.AHGGFSVFAGVGER.T R.VGLTGLTVAEHFR.D R.FTQANSEVSALLGR.I R.DAEGQDVLLFIDNIFR.F R.IPSAVGYQPTLATDLGGLQER.I |
| 69 | ATP synthase CF1 epsilon subunit | *Prunus persica* | 313183828 | 4.99/14 | 5.42/14 | 480 | 78 | 9 | R.QTIEANLALR.R R.QTIEANLALRR.A M.TLNLCVLTPNR.I + Acetyl (Protein N-term) R.IGNNEITVLVNDAEK.G R.LNDQWLTMALMGGFAR.I R.LNDQWLTMALMGGFAR.I + Oxidation (M) R.LNDQWLTMALMGGFAR.I + 2 Oxidation (M) K.GSDIDPQEAQQTLEIAEANLR.E K.EIILSTNSGQIGVLPNHAPVATAVDIGILR.I |
| 94 | hypothetical protein PRUPE_ppa009109mg | *Prunus persica* | 595826717 | 5.43/33 | 5.43/28 | 721 | 44 | 11 | K.IQYGTLCIR.E K.VYVIGEEGILK.E R.ENPGCLFIATNR.D K.LIDGVPETLDLLR.S K.ELELAGYQYLGGPEDGGK.K R.DAVTHLTDAQEWAGGGSMVGAIR.G R.DAVTHLTDAQEWAGGGSMVGAIR.G + Oxidation (M) K.IDLKPGFLMEHDENVGAVVVGFDR.N K.IDLKPGFLMEHDENVGAVVVGFDR.N + Oxidation (M) K.KIDLKPGFLMEHDENVGAV |
| 22 | PREDICTED: protein DJ-1 homolog D-like | *Prunus mume* | 645243188 | 5.28/43 | 5.66/44 | 531 | 33 | 8 | K.AGDTCPTAIR.D R.VVSSGGTFVEADPIDR.C K.APEFLALKEDVIVLVK.Q R.KPIAAICHGLLVLAPAGAVK.G R.KPVASICHGLEILVAAGVLQGK.K R.VLIIIGNYAEDYEVTVPYQTLK.V R.RVLIIIGNYAEDYEVTVPYQTLK.V R.GHNFTLNATFDDIEFDKYDGLVIPGGR.S |
| 55 | Os02g0101500 | *Oryza sativa Japonica Group* | 115443619 | 6.56/42 | 6.83/41 | 172 | 8 | 4 | K.GQTVGVIGAGR.I R.IVEADQFMR.A + Deamidated (NQ) R.IVEADQFMR.A + Deamidated (NQ); Oxidation (M) R.GPVIDEVALVEHLK.A |
| 72 | cytosolic ATP sulfurylase | *Populus tremula x Populus alba* | 340629183 | 6.45/36 | 5.85/44 | 97 | 10 | 3 | K.YNDGLDHYR.L R.INAGANFYIVGR.D R.ENEYLQSLHFNSLR.M |
| 93 | hypothetical protein PRUPE_ppa008220mg | *Prunus persica* | 595797200 | 6.41/37 | 6.68/35 | 235 | 23 | 5 | K.AGHPTYALVR.E R.FFPSEFGNDVDR.V K.AIFNKEEDIGAYTIK.A K.SLGVNFVLGDLYDHESLVK.A K.GDHTNFEVEPSFGVEASALYPDVK.Y |
| 50 | CLPC | *Theobroma cacao* | 590670650 | 6.36/102 | 5.63/77 | 244 | 11 | 8 | K.TAIAEGLAQR.I R.HAQLPEEAR.E K.YRGEFEER.L R.VLELSLEEAR.Q R.VLENLGADPSNIR.T R.GSGFVAVEIPFTPR.A K.LIGSPPGYVGYTEGGQLTEAVR.R R.YTDDALISAAQLSYQYISDR.F |
| **Carbohydrate metabolism** | | | | | | | | | |
| 52 | hypothetical protein PRUPE_ppa002686mg | *Prunus persica* | 596051666 | 5.38/71 | 5.14/60 | 141 | 9 | 4 | K.FSGALDLDTR.I R.GWVGFNVPVSHR.I + Dioxidation (W) R.YGTVPVVHSTGGLR.D R.AQVSGGSGGAQDGASALEDQREEK.G |
| 75 | prunasin hydrolase isoform PH B precursor | *Prunus serotina* | 15723332 | 5.34/58 | 5.18/56 | 192 | 7 | 3 | R.FSISWSR.I + Dioxidation (W) K.ITDGSNGDVAIDQYHR.Y + Deamidated (NQ) K.SLTGSYDYIGVNYYSAR.Y |
| 76 | prunasin hydrolase isoform PH B precursor | *Prunus serotina* | 15723332 | 5.34/58 | 5.34/55 | 244 | 9 | 4 | R.FSISWSR.I + Dioxidation (W) K.GLYDLVLYTK.E K.ITDGSNGDVAIDQYHR.Y + Deamidated (NQ) K.SLTGSYDYIGVNYYSAR.Y |
| 81 | fructose-bisphosphate aldolase | *Phaseolus vulgaris* | 543176784 | 7.63/43 | 5.98/34 | 206 | 10 | 2 | R.LASIGLENTEANR.Q  R.YAAISQDNGLVPIVEPEILLDGEHTIDR.T |
| 32 | fructokinase, putative | *Ricinus communis* | 255585331 | 5.18/35 | 5.13/32 | 117 | 6 | 2 | R.TALAFVTLR.A K.NAGALLSYDPNLR.L + Deamidated (NQ) |
| 24 | LOS2 | *Capsella bursa-pastoris* | 45477377 | 5.45/47 | 6.14/43 | 221 | 13 | 3 | K.VNQIGSVTESIEAVR.M R.SGETEDTFIADLAVGLSTGQIK.T K.YGQDATNVGDEGGFAPNIQENK.E |
| 16 | PREDICTED: xylulose kinase | *Prunus mume* | 645224530 | 6.16/61 | 6.12/58 | 227 | 13 | 5 | K.AVGGALELSQLTGSR.G R.EVEKEFDGASEVR.A K.EHEILPPLPVGFHR.Y K.DSYFLGFDSSTQSLK.A R.YVLENFNGETLEGVNER.E |
| 61 | triosephosphate isomerase | *Genlisea aurea* | 527186354 | 6.9/33 | 5.75/26 | 205 | 10 | 3 | K.FFVGGNWK.C  R.TFEVCFQQLK.A  K.GGAFTGEISVEQIK.D |
| 56 | hypothetical protein PRUPE_ppa006386mg | *Prunus persica* | 595857285 | 6.27/46 | 5.61/36 | 662 | 41 | 12 | R.LTSVFGGAAEPPK.G K.FYGEVTQQMLK.L + Oxidation (M) K.IRDLFEQLIASK.A R.ANNFDLMYEQVK.A R.ANNFDLMYEQVK.A + Oxidation (M) K.ILVIEGLHPMYDQR.V + Oxidation (M) R.DLLDFSIYLDISNEVK.F R.VRDLLDFSIYLDISNEVK.F K.LSDFPGSNNGTGLFQTIVGLK.I + Deamidated (NQ) K.FFNPVYLFDEGST |
| 77 | hypothetical protein PRUPE_ppa003856mg | *Prunus persica* | 595842590 | 5.94/62 | 5.84/58 | 546 | 26 | 10 | K.DMGLDAYR.F R.GDYPQSMR.Y K.GLYDLVLYTK.K K.ERLPNFTEEQSK.S R.FGINYVDYDNGLKR.H R.GPSVWDAFTHNHPEK.I K.ITDGSNGDVAIDQYHR.Y + Deamidated (NQ) R.ALDFMYGWFMDPLTR.G + Oxidation (M) K.SLIGSYDYIGVNYYSAR.Y R.TNFDTLFPGFTFGTATAAYQLEGAANIDGR.G |
| 90 | prunasin hydrolase isoform PH B precursor | *Prunus serotina* | 15723332 | 5.34/58 | 5.68/56 | 198 | 10 | 4 | K.GLYDLVLYTK.E R.AASDWLYVYPK.G K.ITDGSNGDVAIDQYHR.Y + Deamidated (NQ) K.SLTGSYDYIGVNYYSAR.Y |
| 14 | Glucose-6-phosphate isomerase | *Morus notabilis* | 703142229 | 5.83/71 | 5.47/51 | 477 | 12 | 8 | K.GAIANPDEGR.M K.ELGLFLDVSR.V R.SVGALIALYER.A R.FPMFDWVGGR.T R.FPMFDWVGGR.T + Oxidation (M) R.ESITVTVQEVTPR.S K.GSTDQHAYIQQLR.E R.VGFTDEFVAEMEPR.F + Oxidation (M) |
| 80 | PREDICTED: sedoheptulose-1,7-bisphosphatase, chloroplastic-like | *Pyrus x bretschneideri* | 694366289 | 5.86/43 | 5.30/37 | 287 | 13 | 6 | K.LFSPGNLR.A K.LIVNLDDR.T R.FEETLYGSSR.L R.DQVAAAMGIYGPR.T R.DQVAAAMGIYGPR.T + Oxidation (M) K.GFPGTHEFLLLDEGK.W |
| 23 | ADP-glucose pyrophosphorylase alpha subunit IbAGPa2 | *Ipomoea batatas* | 126363757 | 8.02/57 | 5.77/43 | 423 | 14 | 7 | K.IHHSVVGLR.S R.AKPAVPLGANYR.L R.SVLGIILGGGAGTR.L K.KPVPDFSFYDR.S K.IYVLTQFNSASLNR.H K.AMKVDTTILGLDDQR.A K.AMKVDTTILGLDDQR.A + Oxidation (M) |
| 54 | mandelonitrile glucosyltransferase UGT85A19 | *Prunus dulcis* | 157734205 | 5.03/54 | 5.01/46 | 127 | 4 | 2 | R.DYIDGLVR.T R.FETIPDGLPPADADAR.R |
| 83 | Ferredoxin-thioredoxin reductase catalytic chain, chloroplastic -like protein | *Gossypium arboreum* | 728836173 | 7.56/16 | 5.33/15 | 87 | 13 | 3 | K.FSEQYAR.R R.KFSEQYAR.R K.NSLGAPLCPCR.H + Deamidated (NQ) |
| **Photosynthesis** | | | | | | | | | |
| 36 | putative chlorophyll a/b binding protein, partial | *Aralia elata* | 397789264 | 7.82/16 | 5.19/26 | 195 | 20 | 4 | R.ELEVIHSR.W  K.FGEAVWFK.A + Dioxidation (W)  K.KPVASSGSPWYGPDR.V  K.KPVASSGSPWYGPDR.V + Dioxidation (W) |
| 58 | PREDICTED: 28 kDa ribonucleoprotein, chloroplastic-like | *Nelumbo nucifera* | 719976998 | 4.53/36 | 4.35/38 | 201 | 14 | 4 | R.GSRPERPPR.V  R.VNVAEERPR.R  R.GFGFVTMSTVEEAEK.A + Oxidation (M)  R.IYVGNLPWQVDNSR.L + Deamidated (NQ); Dioxidation (W) |
| 60 | PREDICTED: carbonic anhydrase 2, chloroplastic-like isoform X1 | *Prunus mume* | 645249108 | 8.38/36 | 5.61/27 | 164 | 10 | 2 | K.EAVNVSIGNLLSYPFVR.D  R.VCPSHVLNIQPGEAFVVR.T + Deamidated (NQ) |
| 65 | PREDICTED: gamma carbonic anhydrase 1, mitochondrial | *Jatropha curcas* | 802594263 | 6.09/30 | 6.75/31 | 280 | 26 | 6 | K.HAMVAAGALVR.Q + Oxidation (M) R.GSSIWYGCVLR.G + Dioxidation (W) R.RDEEYDSMLGVVR.E R.RDEEYDSMLGVVR.E + Oxidation (M) R.LQGNYYFQEQLSR.H K.APVVDKEAFVAPSASIIGDVQVGR.G |
| 152 | ribulose-1,5-bisphosphate carboxylase/oxygenase large subunit | *Oxydendrum arboreum* | 6561363 | 6.72/51 | 6.71/50 | 210 | 7 | 3 | K.DTNILAAFR.V + Deamidated (NQ) R.EITLGFVDLLR.D K.TFQGPPHGIQVER.D |
| 70 | ribulose-1,5-bisphosphate carboxylase/oxygenase large subunit | *Pteridophyllum racemosum* | 2687489 | 6.09/50 | 6.52/46 | 659 | 21 | 6 | K.DTDILAAFR.V R.EITLGFVDLLR.D K.TFQGPPHGIQVER.D K.GHYLNATANTCEEMIKR.A R.GGLDFTKDDENVNSQPFMR.W R.ELGVPIVMHDYLTGGFTANTSLQHYCR.D |
| 98 | Ribulose bisphosphate carboxylase/oxygenase activase, chloroplastic | *Vigna radiata var. radiata* | 10720249 | 7.57/48 | 5.20/43 | 150 | 5 | 3 | K.FYWAPTR.D K.FYWAPTR.D + Dioxidation (W) K.MCALFINDLDAGAGR.L + Oxidation (M) |
| 100 | Photosystem II stability/assembly factor HCF136, chloroplastic; Flags: Precursor | *Oryza sativa Japonica Group* | 75252730 | 9.02/45 | 5.44/35 | 224 | 7 | 3 | R.GFGILDVGYR.S R.ADGGLWLLVR.G + Dioxidation (W) K.AAVQETVSATLNR.T |
| 37 | Full=Chlorophyll a-b binding protein of LHCII type I, chloroplastic | *Cucumis sativus* | 115768 | 5.14/27 | 5.24/26 | 124 | 21 | 5 | R.ELEVIHSR.W R.WAMLGALGCVFPELLSR.N R.WAMLGALGCVFPELLSR.N + Oxidation (M) R.WAMLGALGCVFPELLSR.N + Dioxidation (W); Oxidation (M) K.GPLENLADHLADPVNNNAWAYATNFVPGK.- |
| 97 | Thylakoid lumenal 15 kDa protein, chloroplast precursor, putative | *Ricinus communis* | 255566450 | 9.58/22 | 5.26/14 | 218 | 23 | 3 | K.GGGPYGSEVTR.G K.GSNITGAEVTMPQNK.L + Oxidation (M) K.LLGASFFDADLTGADLSDADLR.G |
| 64 | ribulose-1,5-bisphosphate carboxylase/oxygenase large subunit, partial (chloroplast) | *Campanula stricta* | 253992219 | 6.34/50 | 6.86/34 | 309 | 10 | 5 | K.AQAETGEIK.G K.DTDILAAFR.V K.LTYYTPDYETK.D K.DDENVNSKPFMR.W R.GGLDFTKDDENVNSKPFMR.W |
| 41 | Carbonic anhydrase, chloroplastic | *Gossypium arboreum* | 728829487 | 6.54/27 | 6.12/27 | 185 | 13 | 2 | K.YAGTGSAVEYAVLHLK.V K.EAVNVSLGNLLSYPFVR.D |
| 40 | D-Ribulose-5-phosphate 3-epimerase | *Coccomyxa subellipsoidea C-169* | 545358118 | 8.49/29 | 6.61/27 | 118 | 5 | 1 | K.DSIIVSPSILSANFAK.L |
| 59 | carbonic anhydrase 2 | *Flaveria bidentis* | 27652184 | 6.01/31 | 5.57/27 | 156 | 12 | 2 | K.EAVNVSLGNLLTYPFVR.E R.VCPSHILNFQPGEAFVVR.N |
| 43 | PREDICTED: ribonucleoprotein At2g37220, chloroplastic | *Prunus mume* | 645277805 | 5.05/29 | 4.44/20 | 266 | 23 | 6 | R.VNYGPPPPR.S R.QLNGYELDGR.A + Deamidated (NQ) R.GFGFVTMSSVQEAEGAAR.Q R.GFGFVTMSSVQEAEGAAR.Q + Oxidation (M) R.GFGFVTYGTADEMNSAIESLDGVDLNGR.S R.GFGFVTYGTADEMNSAIESLDGVDLNGR.S + Oxidation (M) |
| 11 | ribulose-1,5-bisphosphate carboxylase/oxygenase large subunit, partial (chloroplast) | *Sagittaria latifolia* | 169838 | 6.53/44 | 4.80/50 | 55 | 2 | 1 | R.QLTLGFVDLLR.D + Deamidated (NQ) |
| 57 | Photosystem II stability/assembly factor, chloroplast (HCF136) isoform 1 | *Theobroma cacao* | 590689698 | 7.75/45 | 5.46/34 | 208 | 16 | 6 | R.RIQNMGWR.A R.ADGGLWLLVR.G K.GTGITEDFEEIPVQSR.G R.QTILETKDGGNTWVPR.S + Deamidated (NQ) R.GNFYLTWEPGQPFWQPHNR.A R.GNFYLTWEPGQPFWQPHNR.A + Dioxidation (W) |
| 29 | 31 kDa ribonucleoprotein | *Morus notabilis* | 703157211 | 4.68/38 | 4.30/29 | 118 | 9 | 4 | R.VNVAEQRPR.L + Deamidated (NQ)  R.LEQVFSEHGK.V  R.GFGFVTMSTVEEAEK.A  R.GFGFVTMSTVEEAEK.A + Oxidation (M) |
| 74 | photosystem II oxygen-evolving complex protein 2 precursor | *Populus trichocarpa* | 224085421 | 8.65/28 | 5.59/21 | 180 | 15 | 4 | K.HQLITATVK.D K.QYFFLSVLTR.T + Deamidated (NQ) R.KFVESTASSFSVA.- R.TADGDEGGKHQLITATVK.D |
| 82 | 23 kDa OEC protein, partial | *Salicornia veneta* | 148535011 | 5.94/22 | 6.51/22 | 133 | 13 | 3 | K.HQIITATVK.D K.EVEYPGQVLR.F R.TADGDEGGKHQIITATVK.D |
| 102 | PREDICTED: carbonic anhydrase 2, chloroplastic-like isoform X1 | *Prunus mume* | 645249108 | 8.38/36 | 5.75/27 | 60 | 10 | 2 | K.EAVNVSIGNLLSYPFVR.D R.VCPSHVLNIQPGEAFVVR.T + Deamidated (NQ) |
| **Transcription and translation** | | | | | | | | | |
| 21 | eukaryotic initiation factor 4A | *Oryza sativa* | 12381896 | 5.51/47 | 5.50/43 | 90 | 3 | 1 | R.GIYAYGFEKPSAIQKR.G |
| 95 | PREDICTED: 28 kDa ribonucleoprotein, chloroplastic | *Amborella trichopoda* | 586721656 | 4.66/35 | 4.30/25 | 86 | 12 | 2 | K.AGIVEVAEVIYNR.E R.GFGFVTMSTETELNDAIAALDGQSLDGR.A + Deamidated (NQ); Oxidation (M) |
| 67 | eIF5A3 | *Populus deltoides* | 223047854 | 5.48/17 | 5.81/17 | 344 | 45 | 6 | K.TYPQQAGTIR.K  M.SDEEQHFESK.A + Acetyl (Protein N-term)  R.LPTDESLLSQIK.D  K.CHFVAIDIFNGK.K  K.DDLRLPTDESLLSQIK.D  R.TDYQLIDISEDGFVSLLTENGNTK.D |
| 49 | eukaryotic translation initiation factor 5A | *Arachis hypogaea* | 403084315 | 5.60/17 | 6.03/17 | 288 | 50 | 6 | K.TYPQQAGTIR.K R.LPTDDSLLTQIK.D K.CHFVGIDIFTAK.K K.DDLRLPTDDSLLTQIK.D K.KLEDIVPSSHNCDVPHVNR.T R.TDYQLIDISEDGFVSLLTENGNTK.D |
| 4 | OSJNBa0091D06.15 | *Oryza sativa Japonica Group* | 38567873 | 5.69/82 | 5.16/74 | 167 | 7 | 3 | R.VEANVGAPQVNYR.E R.INIIDTPGHVDFTLEVER.A R.AVLVDGSYHDVDSSVLAFQIAAR.G |
| **Transport** | | | | | | | | | |
| 107 | hypothetical protein CICLE_v10022955mg | *Citrus clementina* | 567895834 | 5.39/13 | 5.01/14 | 87 | 26 | 3 | K.LTAPFDFPR.F K.IKYEDFIAR.M K.LEPSEFDEWIR.E |
| 35 | isopentenyl pyrophosphate:dimethyllallyl pyrophosphate isomerase | *Arabidopsis thaliana* | 6856564 | 5.48/30 | 5.08/28 | 119 | 9 | 2 | R.LVVDNFLMK.W + Oxidation (M) R.LMFEDECILVDENNR.V + Deamidated (NQ) |
| **Stress and defense** | | | | | | | | | |
| 30 | PREDICTED: plastid-lipid-associated protein, chloroplastic-like | *Malus domestica* | 657984057 | 5.71/34 | 4.50/31 | 299 | 18 | 4 | K.TISSQPPFK.F R.GDNGSVFVLIK.A K.ALVDSFYGTNR.G + Deamidated (NQ) R.AEIVELITQLEAKNPTPAPTEALPLLNGK.W |
| 86 | PREDICTED: probable glutathione S-transferase | *Prunus mume* | 645239723 | 5.82/25 | 6.35/26 | 468 | 38 | 7 | R.FWADYVDKK.M K.APLLPSDPHLR.A K.SPLLLQSNPVHK.Q K.FGNFSVEAEHPK.F K.DKAPLLPSDPHLR.A M.ADQVVLLDFWPSPFGMR.L + Acetyl (Protein N-term); Dioxidation (W); Oxidation (M) K.EFLECISVLEGELGDKPYFGGK.T |
| 87 | thioredoxin peroxidase, partial | *Nicotiana tabacum* | 407907615 | 8.25/29 | 4.64/22 | 351 | 21 | 4 | K.SYNVLIPDQGIALR.G + Deamidated (NQ) K.SGGLGDLNYPLISDVTK.S R.KSGGLGDLNYPLISDVTK.S R.TLQALQYVQDNPDEVCPAGWKPGEK.S |
| 92 | PREDICTED: universal stress protein A-like protein | *Solanum lycopersicum* | 460395696 | 5.34/24 | 5.55/32 | 90 | 10 | 2 | R.LCLEVER.L  R.VAIAVDLSDESAFAVK.W |
| 104 | peroxiredoxin | *Phaseolus vulgaris* | 11558242 | 5.17/28 | 4.73/27 | 424 | 28 | 5 | K.SYDVLIPDQGIALR.G K.EGVIQHSTINNLAIGR.S K.SGGLGDLNYPLISDVTK.S R.KSGGLGDLNYPLISDVTK.S R.TLQALQYVQENPDEVCPAGWKPGEK.S + 2 Deamidated (NQ); Dioxidation (W) |
| 88 | hypothetical protein PRUPE_ppa012662mg | *Prunus persica* | 595927644 | 5.95/18 | 6.37/20 | 237 | 20 | 2 | K.DVNQPSLVHIK.S K.DQSTAELPNRLECSGEESHSK.Y |
| 38 | abscisic acid stress ripening protein homolog | *Prunus mume* | 292494380 | 5.62/21 | 5.91/31 | 965 | 53 | 8 | K.KDPEHAHR.H K.HHHGLFHHHK.D K.ETAAYGTTGTHESEIDYK.K K.ETAAYGTTGTHESEIDYKK.E K.HLEHLSEAGAAAAGVFALHEK.H K.DEDRPIETSDYPQSGGYSDEGR.T K.IEEEIAAAAAVGSGGFAFHEHHEK.K R.HKIEEEIAAAAAVGSGGFAFHEHHEK.K |
| 101 | PREDICTED: putative lactoylglutathione lyase-like isoform X1 | *Glycine max* | 356531939 | 6.14/33 | 5.52/31 | 109 | 7 | 3 | K.ITSFLDPDGWK.T  K.TVLVDNQDFLK.E + Deamidated (NQ)  K.ITSFLDPDGWK.T + Dioxidation (W) |
| 32 | abscisic stress ripening protein homolog | *Prunus armeniaca* | 2677824 | 5.64/21 | 5.13/32 | 63 | 24 | 2 | K.DEDRPIETSDYPQSGGYSDEGR.T K.HKIEEEIAAAAAVGSGGFAFHEHHEK.K |
| 91 | catalase | *Eleutherococcus senticosus* | 558064716 | 7.11/56 | 6.47/37 | 210 | 10 | 3 | K.TWPEDILPLQPVGR.L R.EGNFDLVGNNFPVFFVR.D K.GFFEVTHDISKLTCADFLR.A |
| 13 | Full=Catalase isozyme 2 | *Cucurbita pepo* | 1345678 | 7.04/57 | 5.45/50 | 137 | 5 | 2 | R.APGVQTPVIVR.F R.EGNFDLVGNNFPVFFVR.D |
| 39 | glutathione S-transferase, amino-terminal domain protein | *Medicago truncatula* | 357494177 | 5.63/25 | 6.86/25 | 204 | 17 | 4 | R.VLVCLVEK.E K.VLNIYEER.L + Deamidated (NQ) K.RVLVCLVEK.E K.LQPFGNVPVIKDGDYTLYESR.A + Deamidated (NQ) |
| 75 | glutathione S-transferase, amino-terminal domain protein | *Medicago truncatula* | 922378835 | 5.74/25 | 5.18/56 | 99 | 13 | 2 | K.VLDIYEER.L K.LQPFGLLPVIQDGDYTLYESR.A + Deamidated (NQ) |
| 45 | hypothetical protein PRUPE_ppa011383mg | *Prunus persica* | 595793483 | 5.77/24 | 6.16/25 | 347 | 32 | 8 | R.VFAALYEK.D K.LAAVLDVYEAR.L K.VYGSTYSTAAMR.V K.VYGSTYSTAAMR.V + Oxidation (M) K.DIEFEFVPVDMR.A K.DIEFEFVPVDMR.A + Oxidation (M) K.MAILSVWTEVEGQK.F R.AITQYIAHEYFDK.G |
| 28 | hypothetical protein PRUPE_ppa008881mg | *Prunus persica* | 595826552 | 6.19/35 | 6.62/35 | 738 | 40 | 11 | K.VLLEIATTR.S K.AYNDEELIR.I K.LLVPLLSTYR.Y R.DAFLANEATKR.F R.AVLLWTLVPAER.D R.AVLLWTLVPAER.D + Dioxidation (W) K.SLDKELSSEFER.A K.ADKDDEFLLLLR.Q K.SLEEDVAYHTSGDLRK.L R.VPDVVPSPVEDAEQLRK.A K.AFEGWGTNEALIISILTHR.N |
| 34 | hypothetical protein PRUPE_ppa009612mg | *Prunus persica* | 596015068 | 7.81/32 | 6.68/32 | 288 | 27 | 5 | R.NLELSEAVK.K K.EGGYGLIIPK.G R.NEETGEINIIYK.R R.AEEEAETLYASIDLASSIIQR.K K.YFDMPPLTVAEAVDQLENVDHDFYAFR.N |
| 31 | quinone oxidoreductase-like family protein | *Populus trichocarpa* | 566168414 | 7.64/34 | 4.79/32 | 219 | 13 | 4 | K.GMTAQFLLR.R + Oxidation (M) R.VHELGGPEVLK.W K.DTFQGSLACLK.T + Deamidated (NQ) K.AIGLNFIDVYFR.K |
| 105 | PREDICTED: 2-Cys peroxiredoxin BAS1, chloroplastic | *Solanum lycopersicum* | 460365357 | 6.18/29 | 4.72/22 | 133 | 11 | 2 | K.SYNVLIPDQGIALR.G + Deamidated (NQ) K.EGVIQHSTINNLAIGR.S |
| 20 | hypothetical protein PRUPE_ppa011383mg | *Prunus persica* | 595793483 | 5.77/24 | 4.94/38 | 271 | 32 | 7 | R.VFAALYEK.D K.LAAVLDVYEAR.L K.VYGSTYSTAAMR.V K.DIEFEFVPVDMR.A K.DIEFEFVPVDMR.A + Oxidation (M) K.MAILSVWTEVEGQK.F R.AITQYIAHEYFDK.G |
| 48 | hypothetical protein PRUPE_ppa012591mg | *Prunus persica* | 596197999 | 6.31/18 | 6.62/16 | 249 | 27 | 3 | K.LLVLGSHGR.G R.ALLGSVSNYCVHNAK.C K.DICANHGIVAEAVTEVGDPK.E |
| 103 | 2-Cys peroxiredoxin, partial | *Nicotiana benthamiana* | 511774224 | 8.51/25 | 4.69/23 | 223 | 20 | 3 | K.SYNVLIPDQGIALR.G + Deamidated (NQ)  K.EGVIQHSTINNLAIGR.S  K.SGGLGDLNYPLISDVTK.S |
| 46 | hypothetical protein PRUPE_ppa012585mg | *Prunus persica* | 595830313 | 6.20/18 | 6.84/18 | 467 | 59 | 9 | K.LDCLVLGSR.G R.NIGVAVDFSK.G K.WAINNLAHEK.G K.GDTLYAIHVKPSQGDESR.N K.LCGAVEDLKLDCLVLGSR.G R.NLLWSTTGSPLIPLEEFR.Q R.NLLWSTTGSPLIPLEEFR.Q + Dioxidation (W) K.AVMENYEVEVDPEVLDLLDTAHR.Q K.AVMENYEVEVDPEVLDLLDTAHR.Q + Oxidation (M） |
| 89 | putative luminal binding protein | *Corylus avellana* | 10944737 | 4.97/74 | 5.68/91 | 224 | 11 | 5 | R.ALSSQHQVR.V K.FDLTGVPPAPR.G R.ITPSWVGFTDGER.L R.VEIESLFDGVDFSEPLTR.A K.SQVFTTYQDQQTTVSIQVFEGER.S |
| 66 | putative glycine-rich RNA-binding protein | *Prunus avium* | 34851124 | 7.82/17 | 6.05/15 | 785 | 72 | 10 | M.ASAEIEFR.C + Acetyl (Protein N-term) R.GSGGSEGGSWR.S R.EGGYGGGEGGGAR.Y R.NITVNEAQSR.G R.GSGGGGGGNGGGYSR.G + Deamidated (NQ) R.GFGFVTFSNEK.A R.AFSPFGEIIESK.I R.GGGGGGYGGGGGYGGGGR.R R.DAIEGMNGQNLDGR.N + Oxidation (M) R.CFVGGLAWATDNDALER.A |
| 17 | PREDICTED: 2-hydroxyacyl-CoA lyase | *Beta vulgaris subsp. vulgaris* | 731315932 | 5.96/61 | 6.18/58 | 106 | 5 | 2 | K.GLIPDTHQLAATAAR.S + Deamidated (NQ) R.KPAVINVIIDPYAGAESGR.M |
| 26 | arginase 2 | *Solanum lycopersicum* | 350538867 | 5.60/36 | 5.80/35 | 193 | 13 | 4 | R.IMEGGYAR.R + Oxidation (M)  R.FGVEQYEMR.T  R.VLTDVGDLPVQELR.D  R.EAIWCGSTNSTTEEGK.I |
| 18 | arginase 2 | *Solanum lycopersicum* | 350538867 | 5.60/37 | 4.84/43 | 154 | 11 | 4 | R.IMEGGYAR.R + Oxidation (M)  R.RLLQVGIR.S  R.FGVEQYEMR.T  R.VLTDVGDLPVQELR.D |
| 33 | hypothetical protein PRUPE_ppa009612mg | *Prunus persica* | 596015068 | 7.81/32 | 6.54/31 | 105 | 16 | 2 | R.AEEEAETLYASIDLASSIIQR.K K.YFDMPPLTVAEAVDQLENVDHDFYAFR.N |
| 31 | PREDICTED: ran-binding protein 1 homolog c-like | *Prunus mume* | 645216461 | 4.76/26 | 4.79/32 | 133 | 39 | 5 | R.FASIENNR.L K.MTVQEHAGNEK.S + Oxidation (M) R.LEEVAVTTGEEDEDAILDLK.A K.SSVWHATDFADGELKEELFCIR.F M.SSADAEHREDEEAPLAGDDEDTGAQVAPIVR.L + Acetyl (Protein N-term) |
| **Molecular chaperones** | | | | | | | | | |
| 2 | PREDICTED: endoplasmin homolog | *Beta vulgaris subsp. Vulgaris* | 731360552 | 4.90/92 | 5.08/88 | 188 | 5 | 4 | K.LGIIEDAANR.N  K.LTSLDQYISR.M R.NSAQKFEFQAEVSR.L R.RVFISDEFDELLPK.Y |
| 106 | Full=17.8 kDa class I heat shock protein | *Daucus carota* | 123548 | 7.90/17 | 5.95/16 | 94 | 15 | 2 | K.VLQISGER.N  K.ETPQAHVFKADLPGLK.K |
| 109 | small molecular heat shock protein 19 | *Nelumbo nucifera* | 125620180 | 6.23/19 | 6.02/18 | 107 | 10 | 2 | R.VLQISGER.K R.QFRLPENAK.V |
| 1 | PREDICTED: heat shock 70 kDa protein 15-like | *Prunus mume* | 645244630 | 5.21/95 | 5.38/104 | 479 | 14 | 10 | K.IDVFQNAR.A R.QFSDPVVQR.D K.YQEFVTEPER.E K.YANPVLLSADVR.R K.ILAHSFDQSLGGR.D R.DEFEQISVPILER.V K.RDEFEQISVPILER.V M.SVVGFDFGNESCIVAVAR.Q + Acetyl (Protein N-term) K.SLPFAVTEGPDGYPLIHAR.Y K.GSGPDTQNGAADNNQSTIVFPK.G + Deamidated (NQ) |
| 3 | Endoplasmin-like protein | *Morus notabilis* | 703081280 | 5.08/92 | 4.90/79 | 107 | 3 | 2 | K.FGWSANMER.L  R.FLSVTEPSLLGDAGQLEIR.I |
| 9 | Os01g0840100 | *Oryza sativa Japonica Group* | 115440955 | 5.10/71 | 5.22/62 | 539 | 17 | 9 | K.DAGVIAGLNVMR.I R.VEIIANDQGNR.T K.NALENYAYNMR.N K.NALENYAYNMR.N + Oxidation (M) K.SSVHDVVLVGGSTR.I R.TTPSYVAFTDSER.L R.ARFEELNMDLFR.K K.NAVVTVPAYFNDSQR.Q R.TLSSTAQTTIEIDSLYEGIDFYSTITR.A |
| 5 | RecName: Full=Calreticulin; Flags: Precursor | *Prunus armeniaca* | 11131904 | 4.40/49 | 4.44/52 | 123 | 15 | 5 | K.YVGIELWQVK.S R.FYAISAEFPEFSNK.D R.FYAISAEFPEFSNKDK.T K.SGTLFDNILITDEPEYAK.Q K.APLIDNPEFKDDPELYVYPNLK.Y |
| 10 | luminal binding protein (BiP) | *Arabidopsis thaliana* | 1303695 | 5.08/73 | 5.24/66 | 482 | 15 | 8 | K.DAGVIAGLNVAR.I K.FDLTGVPPAPR.G R.FEELNNDLFR.K K.NGHVEIIANDQGNR.I K.DAVVTVPAYFNDAQR.Q K.IKDAVVTVPAYFNDAQR.Q K.LKEVEAVCNPIITAVYQR.S K.SQVFTTYQDQQTTVSIQVFEGER.S |
| **Other Materials Metabolism** | | | | | | | | | |
| 7 | Full=(R)-mandelonitrile lyase 4 | *Prunus serotina* | 62900446 | 4.86/61 | 4.88/61 | 340 | 11 | 4 | R.FVSEDGIDNVR.S R.ILGGTTIINAGVYAR.A R.DTVASYWHYHGGAIVGK.V R.VVDGSTFPATPASHPQGFYLMLGR.Y + Oxidation (M) |
| 44 | allene oxide cyclase | *Camptotheca acuminata* | 61808301 | 8.71/27 | 5.56/20 | 208 | 11 | 3 | K.LLYTFYLK.G K.LHQIVFPFK.L K.VQELHVYEINER.D |
| 39 | PREDICTED: peptide methionine sulfoxide reductase A1-like | *Nicotiana tomentosiformis* | 697100910 | 8.44/29 | 6.89/28 | 242 | 21 | 5 | R.HDPTTLNR.Q R.QGNDVGTQYR.S R.AEEYHQQYLAK.G R.SGIYFYTPEQEK.A K.ECNFDTLLDVFWDR.H + Dioxidation (W) |
| 96 | hypothetical protein PRUPE_ppa011916mg | *Prunus persica* | 595807521 | 5.06/22 | 5.05/24 | 60 | 18 | 2 | K.LNPHPEGGFYFETFR.D K.VERPVSSSIYFLLPSGDVSR.L |
| **Unknown function** | | | | | | | | | |
| 19 | uncharacterized protein LOC100798637 | *Glycine max* | 363807032 | 4.98/47 | 5.14/44 | 346 | 21 | 7 | R.ADILDPALMR.S K.GVLLYGPPGTGK.T K.RFDSEVSGDR.E K.AVCVEAGMLALR.R + Oxidation (M) R.KIEFPHPTEDAR.A K.LAGPQLVQMFIGDGAK.L + Oxidation (M) R.TMLELLNQLDGFSSDDR.I + Oxidation (M) |
| 62 | hypothetical protein PRUPE_ppa008139mg | *Prunus persica* | 595825894 | 6.18/38 | 6.11/39 | 285 | 13 | 5 | K.LDIYLLR.K K.NVDQLIHR.I K.NSSGAVTALLR.W K.VGLFPDVLER.K K.NSGESNDELFR.A |
| 73 | APE1 | *Zea mays* | 195639368 | 9.06/28 | 5.47/21 | 173 | 8 | 2 | K.YSLVLVFQAK.A + Deamidated (NQ)  R.YGDEQHLDEALQR.I |

^a^ Spot number indicated in Fig. 6; ^b^ Database accession numbers according to the NCBIInr; ^c^ Theoretical and ^d^ experimental masses (kDa) and p*I* of identified proteins; ^e^ Mascot protein score reported after searching against the NCBInr database; ^f^ (%), percentage of the covered region of the identified peptide sequences in the matched protein; ^g^ Number of peptides sequenced.
